# An in silico approach to analyze HCV genotype-specific binding-site variation and its effect on drug–protein interaction

**DOI:** 10.1038/s41598-020-77720-9

**Published:** 2020-11-30

**Authors:** Ramsha Khalid, Muhammad Faraz Anwar, Muhammad Aanish Raees, Sadaf Naeem, Syed Hani Abidi, Syed Ali

**Affiliations:** 1 Department of Biochemistry, University of Karachi, Karachi, Pakistan; 2 Department of Biological and Biomedical Sciences, Aga Khan University, Karachi, Pakistan; 3 Cincinnati Children’s Hospital Medical Center, Ohio, USA; 4 Department of Biological Sciences, Nazarbayev University School of Medicine, Nazarbayev University, Astana, Kazakhstan

**Keywords:** Virtual drug screening, Hepatitis C virus

## Abstract

Genotype variation in viruses can affect the response of antiviral treatment. Several studies have established approaches to determine genotype-specific variations; however, analyses to determine the effect of these variations on drug–protein interactions remain unraveled. We present an in-silico approach to explore genotype-specific variations and their effect on drug–protein interaction. We have used HCV NS3 helicase and fluoroquinolones as a model for drug–protein interaction and have investigated the effect of amino acid variations in HCV NS3 of genotype 1a, 1b, 2b and 3a on NS3-fluoroquinolone interaction. We retrieved 687, 667, 101 and 248 nucleotide sequences of HCV NS3 genotypes 1a, 1b, 2b, and 3a, respectively, and translated these into amino acid sequences and used for genotype variation analysis, and also to construct 3D protein models for 2b and 3a genotypes. For 1a and 1b, crystal structures were used. Drug–protein interactions were determined using molecular docking analyses. Our results revealed that individual genotype-specific HCV NS3 showed substantial sequence heterogeneity that resulted in variations in docking interactions. We believe that our approach can be extrapolated to include other viruses to study the clinical significance of genotype-specific variations in drug–protein interactions.

## Introduction

Genetic variability in the viral population can greatly accelerate the rate of viral evolution in response to different selection pressures^1,2^. Therefore, the characterization of genomic variations in viral variants is essential to understand various aspects of evolution, persistence, epidemiology, immune escape, and development of antiviral drug resistance^1–4^. Additionally, this analysis can identify crucial variations/mutations associated with variability in antiviral treatment response.

Hepatitis C virus (HCV) can serve as a model to study genotype-specific mutations as the clinical significance of HCV genotypes in treatment response is well established^5^. Studies have suggested that genetic variability in HCV genotypes can greatly affect T-cell mediated immune response^6,7^. Furthermore, the sequence heterogeneity in the HCV genome leads to the generation of HCV genotype-specific epitopes that can potentially evade immune surveillance^6,7^. Genotypic variation is also known to play a role in determining the course of disease progression and antiviral response^6^. In the recent past, the combination of pegylated interferon-alpha and a nucleotide analog ribavirin-based regimen was the standard care of treatment for HCV infection. HCV genotype 1 was considered to be the most challenging genotype to treat with this regimen. This regimen gave sustained virologic responses (SVRs) in 40%-50% of HCV genotype 1 infected patients as compared to 75%-80% in HCV genotype 2 and 3 infected patients^5^.

In this paper, we present an in-silico approach to explore genotype-specific amino acid variations and their effect on drug–protein interaction. In our study, HCV NS3 helicase was used as a model drug target whereas fluoroquinolones were used as model drugs. HCV NS3 is a multifunctional enzyme that belongs to DExH Box RNA helicases of superfamily 2 and exhibits NTP-mediated nucleic acid unwinding activity. NS3 plays a pivotal role in HCV biology particularly in the viral replicative cycle, viral assembly, persistence, and pathogenesis^8–10^. Additionally, NS3 is well characterized in terms of structure, domains, functionally active amino acid residues, nucleic acid interaction and unwinding mechanism. However, the genotype-specific variations in HCV NS3 and their effect on NS3 structure, and subsequently on drug–protein interactions, are relatively unexplored.

Fluoroquinolones are broad-spectrum antibiotics that target DNA gyrase and topoisomerase IV—bacterial enzymes involved in DNA replication—forming an irreversible drug–protein-nucleic acid complex^11^. Previously reported studies from our group and others have demonstrated that fluoroquinolones actively inhibit HCV replication by targeting viral protein NS3helicase. Since bacterial gyrase and viral helicase are functionally homologous; it has been hypothesized that fluoroquinolones inhibit viral helicases in a similar fashion^12–15^. To explore how genotype-specific variations in HCV NS3 might affect the inhibition of its activity by fluoroquinolones, we have investigated the effect of active site residue variations in HCV genotype 1a, 1b, 2b and 3a on NS3-fluoroquinolone interactions.

## Results

### Inter-genotype sequence comparison

To explore the inter-genotype sequence similarity in NS3 protein, multiple sequence alignment was performed using NS3 sequences from HCV genotypes 1a, 1b, 2b, and 3a, followed by the construction of sequence identity matrix. The analysis was carried out in two different ways, one using the full-length protein sequence (Table 1a) and the other employing only the fluoroquinolone binding region sequence (Table 1b). Both the approaches revealed an overall high degree of sequence similarity between NS3 helicases from genotypes 1a and 1b, i.e. 93.4% in the full-length protein sequence (Table 1a) and 94% in the fluoroquinolone binding region site (Table 1b). When compared to genotypes 2b and 3a, genotypes 1a and 1b revealed sequence identity scores ranging from 81.5–82.9% for full length protein sequence (Table 1a) and 83.3–88.1% for fluoroquinolone binding region site (Table 1b). Between genotypes 2b and 3a, sequence identity of 80.8% and 85.3% were observed, respectively, for full length and fluoroquinolone binding region site sequences (Table 1a and b).

**Table 1:** Identity matrix of amino acid sequences of NS3 HCV of genotype 1a, 1b, 2b and 3a: Sequence identity was estimated in full length and fluoroquinolone binding region sequences by constructing an ID matrix using the ‘BioEdit’ software. The identity scores are expressed as percentages.

HCV genotypes	1a	1b	2b	3a
**Full length**				
1a	–	93.4	82.1	81.5
1b	93.4	–	82.9	82.8
2b	82.1	82.9	–	80.8
3a	81.5	82.8	80.8	–
**Fluoroquinolone binding region**				
1a	–	94.0	87.3	83.3
1b	94.0	–	88.1	85
2b	87.3	88.1	–	85.3
3a	83.3	85.0	85.3	–

**Figure 1:**
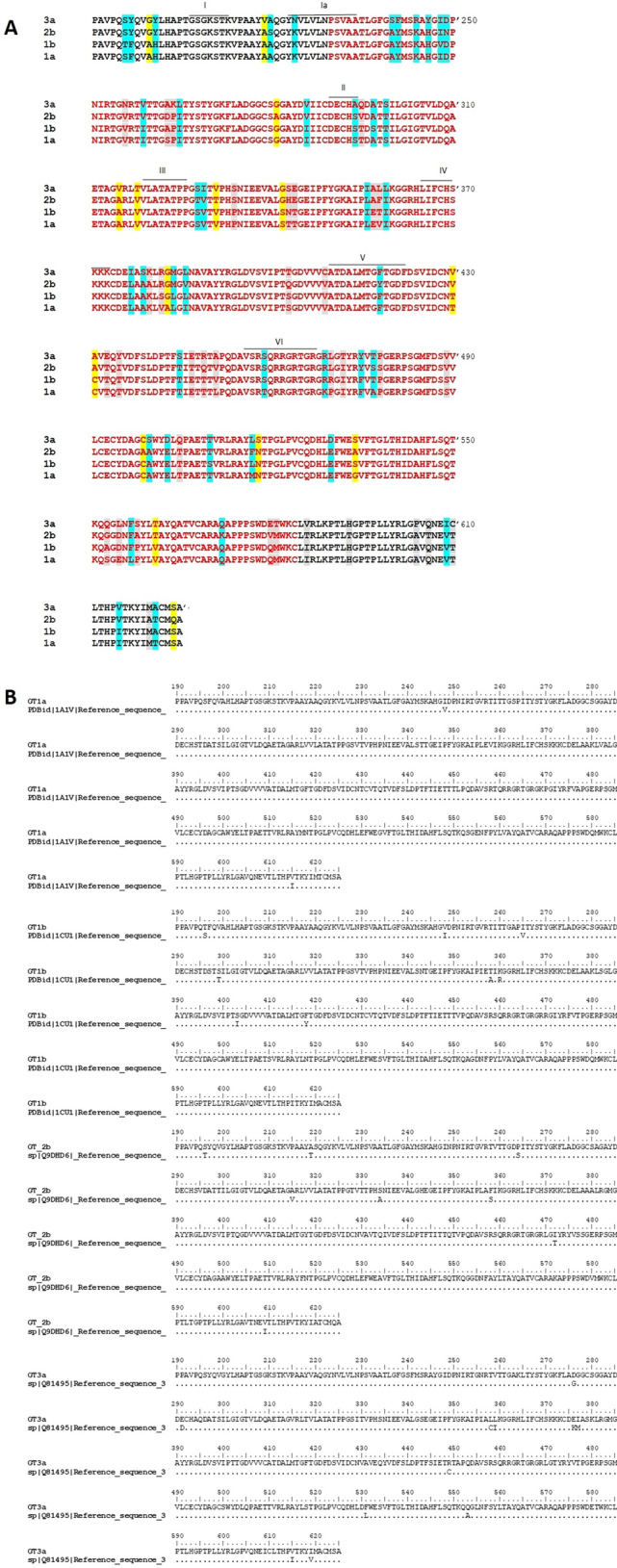
Sequence alignment of HCV NS3 genotypes 1a, 1b, 2b, 3a: (**A**) Multiple sequence alignment was performed employing ‘Clustal W’ using the amino acid sequence in the fluoroquinolone binding region (Pro230-Cys584). The fluoroquinolone binding region is highlighted in red font. Conservative helicase motifs (I-VI) are indicated by black lines on top of the alignment. Conservative, semi-conservative and non- conservative amino acid variations are marked in blue, yellow and grey colors, respectively. (**B**) Pairwise sequence alignment was performed employing ‘Clustal W’ using 1a, 1b, 2b, 3a amino acid sequence and their respective reference sequences 1a (PDB ID:1A1V), 1B (PDBid:1CU1), 2B (UNIPROT ID: Q9DHD6) and 3A (UNIPROT ID: Q81495).

Before inter-genotype mutation analysis (Fig. 1A), the genotype-specific consensus sequences were compared with the available reference sequences. The analysis revealed that the consensus and reference sequences were identical, with exception of 1–2 sites (Fig. 1B), validating that the consensus sequences used in our study represent ‘true’ genotype-specific sequences.

**Figure 2:**
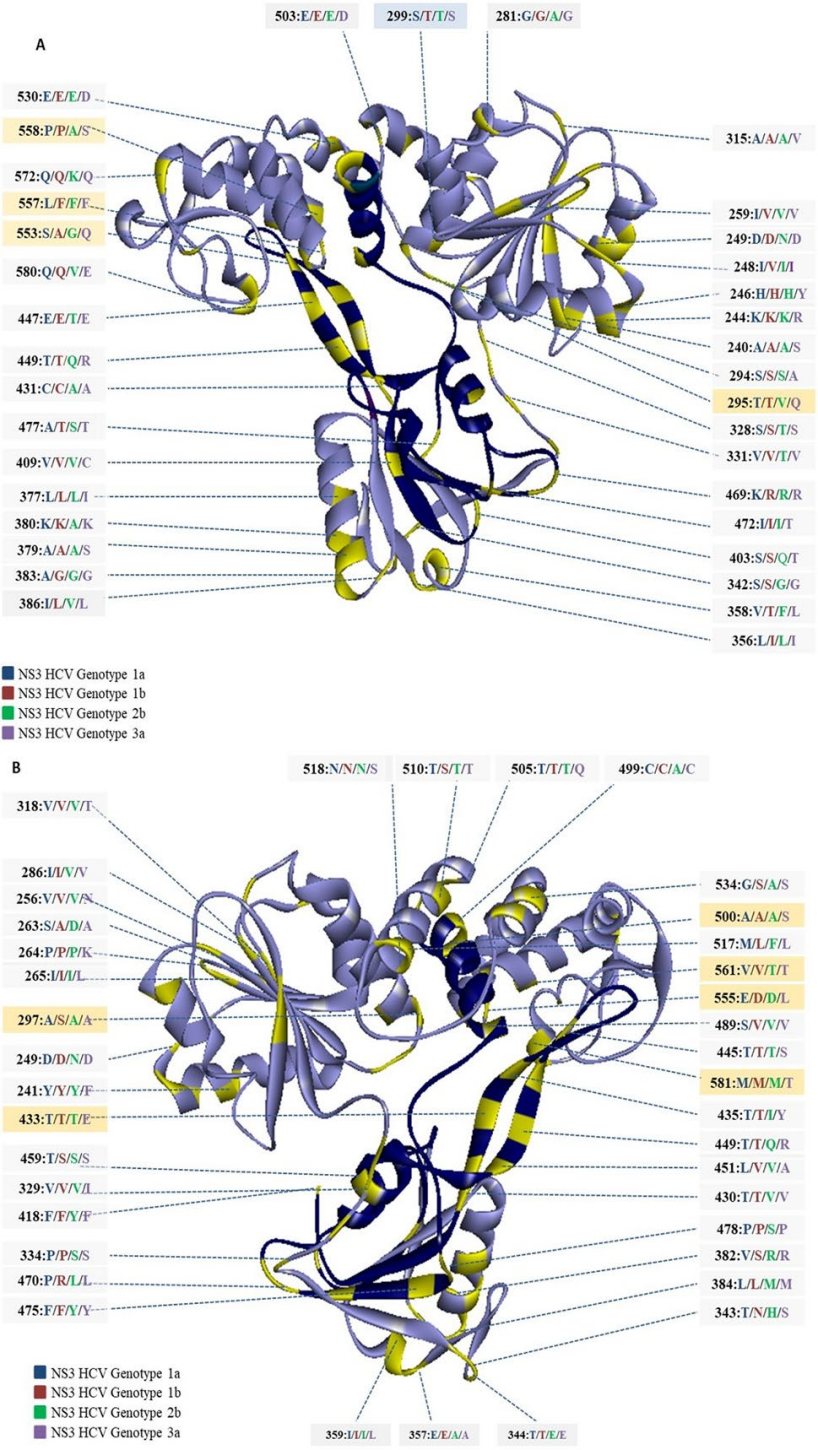
Structure of HCV NS3 with genotype-specific amino acid substitutions across HCV variants 1a, 1b, 2b, and 3a**.** Ribbon Diagram of (**A**) front view, and (**B**) posterior view of NS3 reference structure (PDB ID: 1A1V). Within the fluoroquinolone binding region (P230-C584), amino acid residues variable across HCV genotypes are highlighted in yellow color, while the substrate-binding site, R393-W501, is shaded dark blue. Amino acids of genotype1a, 1b, 2b, and 3a are shown in the blue, red, green and purple font, respectively. A total of 73 amino acid variations were observed. To improve visualization, both the front and posterior views of NS3 were examined. Amino acids that were found to interact with fluoroquinolones are shown in orange boxes.

Mutational analysis of the fluoroquinolone binding region from genotypes 1a, 1b, 2b, and 3a, revealed a total of 90 and 73 variable sites, where genotype 3a revealed the most genotype-specific variations (Figs. 1A and 2). Fluoroquinolone binding region of NS3 sequences contained 33 conservative, 12 semi- conservative and 28 non- conservative amino acid substitutions amongst the four genotypes. Out of these, 3 positions namely 343 T/N/H/S (in genotype 1a/1b/2b/3a), 358V/T/F/L and 553S/A/G/Q showed variable amino acids across all genotypes (Figs. 1A and 2).

Our analysis revealed a high degree of conservation in helicase signature motifs across the four genotypes, except for motif V and motif VI (Fig. 1A), wherein certain genotypes a single amino acid substitution was observed (Fig. 1A). In motif V, F418Y variation was noted, where Y variant was found in genotype 2b; similarly, in motif VI, T459S was observed in genotype 1b, 2b, 3a, while K469R was observed in genotype 1b, 2b, 3a (Figs. 1A and 2). As mentioned in Methods, for this and other analyses 1A1V was used as the reference sequence.

### Validation of 3D structures and docking strategy and genotype-specific variations in NS3-fluoroquinolone interactions

**Figure 3:**
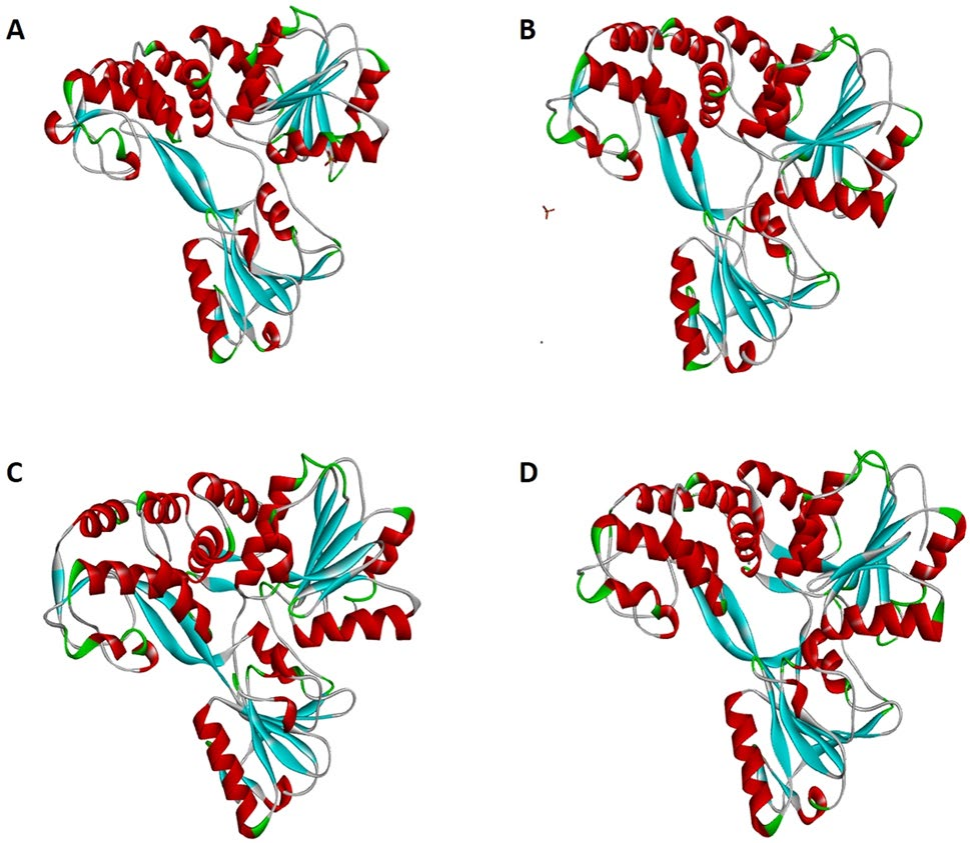
Structures of NS3 HCV: Structures of HCV NS3 used in the study are given. Sutures for (**A**) 1a (PDB ID: 1A1V) and (**B**) 1B (PDBid:1CU1) were retrieved from the PDB database, while structures of (**C**) 2b and (**D**) 3a were constructed using homology modeling approach using CPH model, Swiss model, and Phyre 2 software. Models shown in this figure were constructed using Swiss model.

Before docking the NS3 structures were verified and validated using Verify 3D, GROMACS, and Ramachandran plot analysis. Structures constructed from three different tools were comparable, where structures constructed using Phyre 2 and Swiss-Model gave the lowest RMSD values of 0.41 and 0.0, respectively for genotype 2b and 0.42, and 0.07 for genotype 3a, respectively, when compared to the template structure. CPH tool has higher RMSD values of 1.55 and 1.53 for genotypes 2b and 3a, respectively, when compared to the template structures. All structures passed the 3D verification (performed using Verify 3D software) as at least 80% of the amino acids have scored >  = 0.2 in the 3D/1D profile. Similarly, the structures were valid on the Ramachandran plot as most of the amino acids were under the permissible regions (Supplementary Fig. 1). Based on the lowest RMSD values, overall better quality (based on Verify 3D and Ramachandran plot assessment) models developed using Swiss-Model was used in further analyses (Fig. 3). The structural comparison revealed the structures of the four genotypes to be quite similar (Fig. 3). The RMSD values between structures of genotype 1a–1b, 1a–2b, 1a–3a, 1b–2b, 1b–3a, 2b–3a were found to be 0.01, 0.03, 0.03, 0.03, 0.03, and 0.04, respectively, suggesting strong homology between the NS3 structures from the four genotypes.

**Figure 4:**
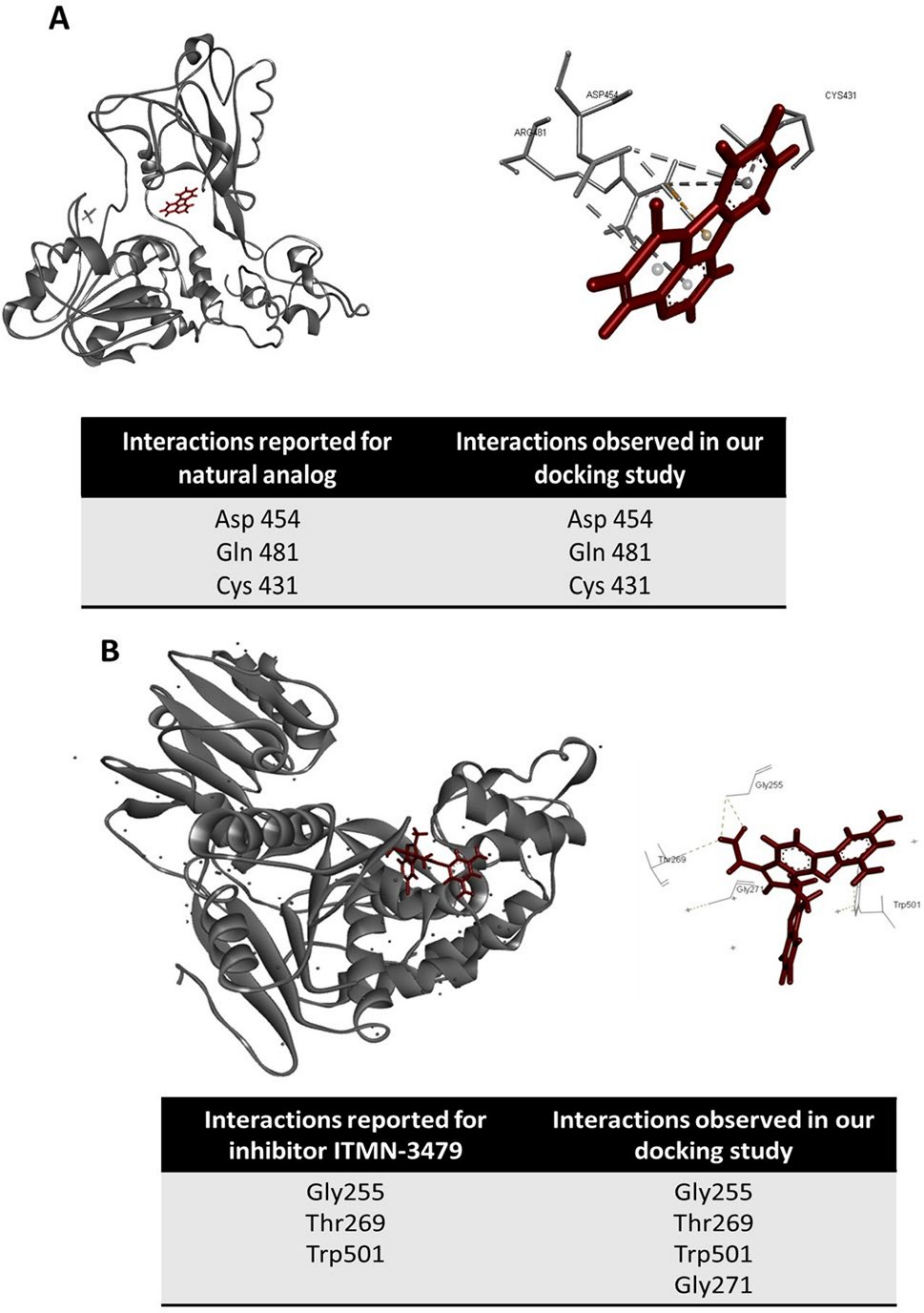
Validation of Docking strategy: Docking strategy was validated by re-docking the two previously published ligands (**A**) natural analog and (**B**) inhibitor ITMN-3479 on their respective ligands. (**A**) and **(B**) Poses of ligands bound to their receptors are shown, while tables at the bottom show amino acid interactions reported for each ligand and observed in our study. Ligand is shown in red, while protein is shown in dark grey color.

Similarly, validation of the docking approach was done by performing blind docking (assuming drug binding site to be anywhere on the protein) on two previously reported complexes of NS3 bound to inhibitors, one a natural analog and other inhibitor ITMN-3479. Our results revealed that for both the models Molegro predicted the exact binding site/pose and gave the similar drug–protein interaction (for natural analog: Asp 454, Gln 481 and Cys 431; for ITMN-3479: Gly255, Thr269, Trp501) as reported for the reference models (Fig. 4 A and B).

To investigate the effect of genotype-specific variations on NS3-fluoroquinolones interactions, molecular docking simulations were performed. Eight fluoroquinolones were docked individually on the consensus NS3 helicase structure constructed for each genotype.

**Figure 5:**
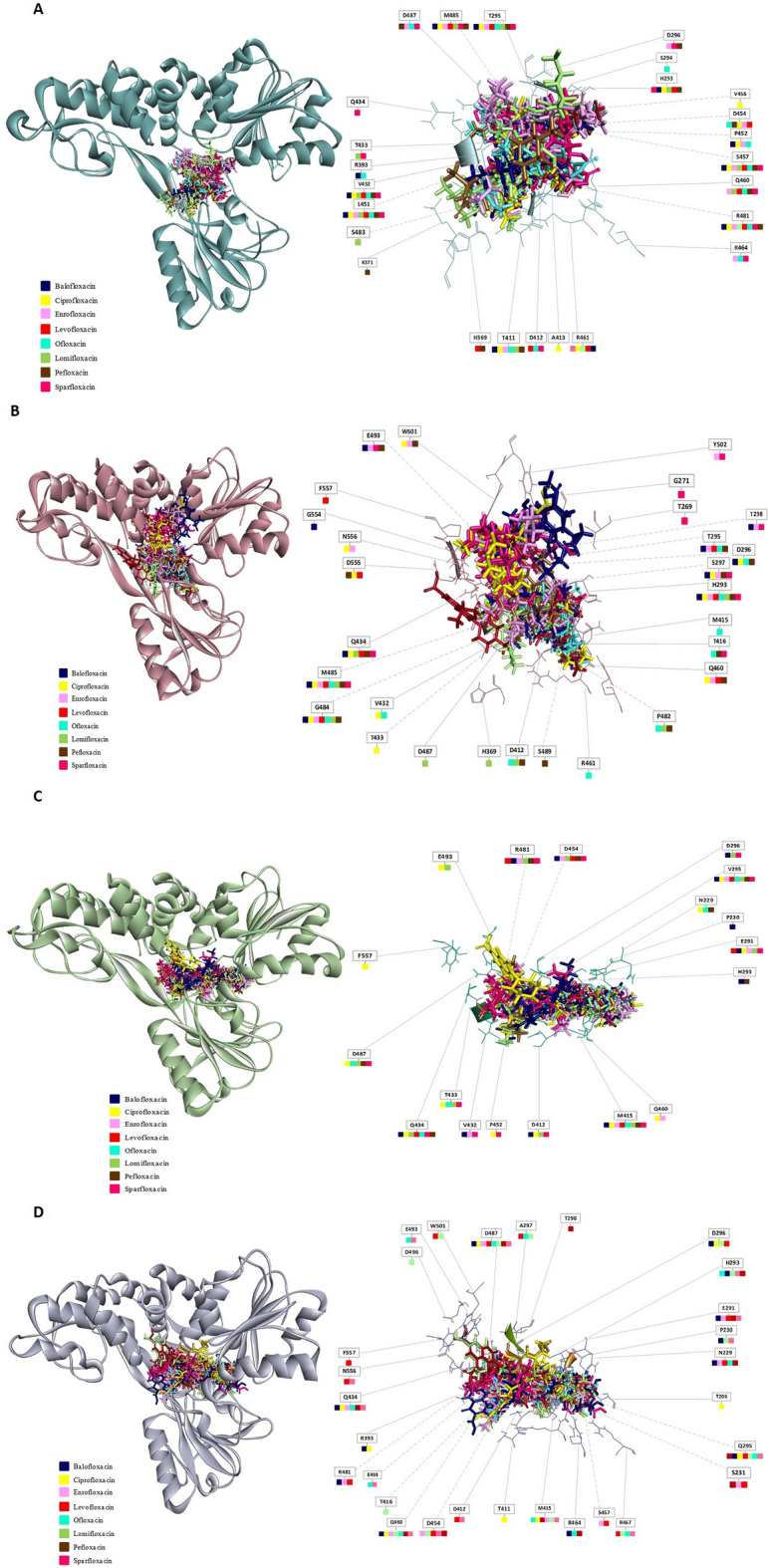
Molecular docking simulation of HCV NS3 with fluoroquinolones constructed for four HCV genotypes**.** HCV NS3 protein each from genotype (**A**) 1a, (**B**) 1b, (**C**) 2b, and (**D**) 3a, was docked with the batch of 8 fluoroquinolones using the Molegro software. In the left panels, a cluster of 8 fluoroquinolones is shown docked into the NS3 binding site, where structures of genotype 1a, 1b, 2b, and 3a are represented with blue, red, green and purple, respectively. The right panels give schematic representations of the fluoroquinolone-interacting amino acids. Binding of fluoroquinolones with each of the amino acids is indicated with color-coded squares where red, purple, light green, dark green, yellow, blue, orange and pink represents Ciprofloxacin, Lomefloxacin, Enrofloxacin, Levofloxacin, Ofloxacin, Pefloxacin, Sparfloxacin, and Balofloxacin, respectively. The grey dashed lines show amino acids located in the back of the fluoroquinolone cluster.

**Figure 6:**
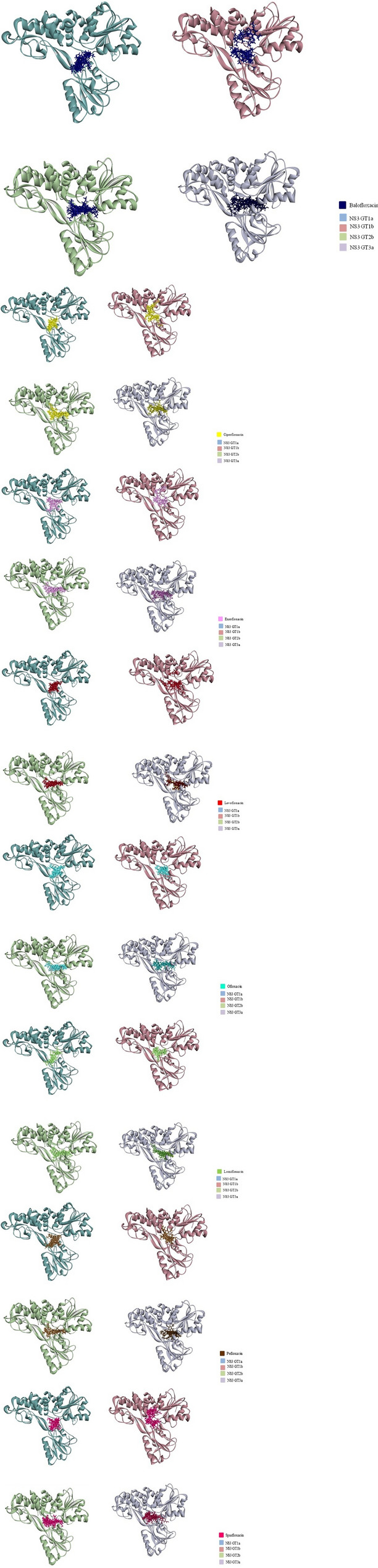
Docking poses of HCV genotype-specific NS3 models interacting with different fluoroquinolones: The structures of NS3 from genotype 1a, genotype 1b, genotype 2b, and genotype 3a are shown in blue, purple, green, and grey, respectively. The docked poses of ligands (top 10 poses) are shown in the following order: (1) Ciprofloxacin, (2) Levofloxacin, (3) Ofloxacin, (4) Pefloxacin, (5) Balofloxacin, (6) Enrofloxacin, (7) Sparfloxacin, and (8) Lomefloxacin.

Comparative analysis of the drug–protein interactions and binding poses revealed that almost all fluoroquinolones interacted with NS3 from all genotypes essentially in the same binding pocket, comprising amino acids Pro230-Cys584 (Figs. 5 and 6; Table 2; Supplementary Table 2). However, in some of the poses, fluoroquinolones, such as Balofloxacin, Ciprofloxacin, and Levofloxacin, etc., exhibited a more scattered interacted with NS3 from genotype 1b (Fig. 6). No amino acid was found to form interactions with all fluoroquinolones, however, certain amino acids appeared to be crucial in the drug–protein interaction (Table 2; Supplementary Table 2). The amino acid residues that frequently made interactions with most fluoroquinolones were Arg481, Asp454, His293, and Gln434 (Table 2). Position 295 in all genotypes appeared to be important in drug–protein interaction as amino acids at this position, despite inter-genotype variation (genotypes 1a and 1b: Thr295; genotype 2b: Val295; genotype 3a: Gln295), interacted with almost all fluoroquinolones (Figs. 5 and 6; Table 2). In addition to position 295, certain genotype-specific residues commonly interacted with each of the fluoroquinolones; these residues include Leu451, Ser457, Asp454, and Arg481 in genotype 1a, and Glu493, and Ser297 in 1b. In contrast, for genotypes 2b and 3a, different residues were involved in forming interactions with different fluroquinolones (Figs. 5 and 6; Table 2; Supplementary Table 2).

**Table 2:** HCV genotype 1a, 1b, 2b and 3a NS3 amino acids residues interacting with Fluoroquinolones: Molegro Virtual Docker software was employed to dock the panel of 8 fluoroquinolones (Sparfloxacin, balofloxacin, Enrofloxacin, Ofloxacin, Pefloxacin, Lomefloxacin, Levofloxacin, and Ciprofloxacin) on each of the four NS3 helicases from different HCV genotypes. The table shows amino acid residues (bold) in the fluoroquinolone binding region of NS3 helicase from different genotypes that formed interactions with the fluoroquinolones. Absence of a reside in genotype(s) is indicated by ‘–’.

Drugs	1a	1b	2b	3a
Balofloxacin	–	–	–	ASN229
	–	–	PRO230	PRO230
	–	–	GLU291	GLU291
	HIS293	HIS293	HIS293	HIS293
	THR295	THR295	VAL295	GLN295
	–	ASP296	ASP296	ASP296
	–	SER297	–	–
	–	THR298	–	–
	ARG393	–	–	ARG393
	THR411	–	–	–
	–	–	ASP412	–
	–	–	MET415	–
	VAL432	–	VAL432	–
	–	–	–	GLU433
	–	GLN434	GLN434	GLN434
	LEU451	–	–	–
	PRO452	–	–	–
	–	ASP454	–	–
	SER457	–	–	–
	–	–	–	GLN460
	–	–	–	ARG464
	–	–	–	ARG467
	ARG481	ARG481	ARG481	–
	GLY484	GLY484	–	–
	MET485	MET485	–	–
	–	–	–	ASP487
	–	GLU493	–	–
	–	GLY554	–	–
Ciprofloxacin	–	–	–	THR206
	–	–	ASN229	–
	–	GLU291	–	–
	HIS293	HIS293	–	–
	THR295	–	VAL295	GLN295
	ASP296	ASP296	–	ASP296
	–	SER297	–	–
	–	–	–	ARG393
	THR411	–	–	THR411
	–	–	ASP412	–
	ALA413	–	–	–
	–	–	MET415	MET415
	VAL432	VAL432	–	–
	–	THR433	THR433	–
	–	GLN434	GLN434	GLN434
	LEU451	–	–	–
	PRO452	–	PRO452	–
	ASP454	–	–	–
	VAL456	–	–	–
	SER457	–	–	–
	–	GLN460	GLN460	GLN460
	ARG461	–	–	–
	–	–	–	ARG467
	ARG481	–	–	–
	GLY484	GLY484	–	–
	MET485	MET485	–	–
	–	–	ASP487	ASP487
	–	–	GLU493	–
	–	TRP501	–	–
	–	ASP555	–	–
	–	ASN556	–	–
	–	–	PHE557	–
Enrofloxacin	–	–	–	ASN229
	–	–	–	SER231
	–	–	GLU291	GLU291
	–	HIS293	–	–
	THR295	THR295	VAL295	–
	–	SER297	–	–
	–	THR298	–	–
	THR411	–	–	–
	–	–	MET415	MET415
	–	–	VAL432	–
	–	–	–	GLU433
	–	–	–	GLN434
	LEU451	–	–	–
	PRO452	–	–	–
	ASP454	ASP454	ASP454	ASP454
	SER457	–	–	SER457
	GLN460	GLN460	GLN460	GLN460
	ARG464	–	–	–
	ARG481	ARG481	ARG481	ARG481
	–	GLY484	–	–
	MET485	MET485	–	–
	ASP487	–	–	ASP487
	–	GLU493	–	–
	–	TRP501	–	–
	–	TYR502	–	–
	–	ASN556	–	–
Lomifloxacin	–	–	–	PRO230
	–	–	GLU291	–
	HIS293	HIS293	–	HIS293
	THR295	–	VAL295	GLN295
	–	–	ASP296	ASP296
	–	–	–	ALA297
	–	HIS369	–	–
	THR411	–	–	–
	–	ASP412	ASP412	–
	–	MET415	MET415	–
	–	–	–	THR416
	VAL432	–	–	–
	THR433	–	THR433	–
	–	GLN434	GLN434	–
	LEU451	–	–	–
	–	ASP454	ASP454	ASP454
	SER457	–	–	–
	–	–	–	GLN460
	ARG461	–	–	–
	–	PRO482	–	–
	SER483	–	–	–
	GLY484	–	–	–
	MET485	MET485	–	–
	ARG481	ARG481	ARG481	–
	–	ASP487	ASP487	ASP487
	–	–	GLU493	–
	–	–	–	ASP496
	–	–	–	TRP501
	–	–	–	SER558
Levofloxacin	–	–	–	ASN229
	–	–	–	SER231
	–	–	GLU291	GLU291
	HIS293	HIS293	–	–
	THR295	THR295	VAL295	GLN295
	–	–	–	ASP296
	–	–	–	ALA297
	HIS369	–	–	–
	ASP412	–	–	ASP412
	–	–	MET415	–
	VAL432	–	–	–
	–	–	THR433	GLU433
	–	GLN434	GLN434	–
	LEU451	–	–	–
	ASP454	–	ASP454	ASP454
	–	ASP555	–	–
	SER457	–	–	SER457
	GLN460	GLN460	–	–
	ARG461	–	–	–
	ARG481	ARG481	ARG481	ARG481
	GLY484	GLY484	–	–
	MET485	MET485	–	–
	–	–	–	ASP487
	–	–	–	TRP501
	–	–	–	ASN556
	–	PHE557	–	PHE557
	–	–	–	SER558
Ofloxacin	–	–	ASN229	ASN229
	–	HIS293	–	HIS293
	SER294	–	–	–
	THR295	THR295	VAL295	GLN295
	–	ASP296	–	–
	–	–	–	ALA297
	THR411	–	–	
	ASP412	ASP412	–	–
	–	MET415	MET415	MET415
	–	THR416	–	–
	VAL432	VAL432	–	–
	–	–	THR433	–
	–	–	GLN434	GLN434
	LEU451	–	–	–
	PRO452	–	–	–
	ASP454	–	–	–
	SER457	–	–	–
	QLN460	–	–	GLN460
	–	ARG461	–	–
	ARG464	–	–	ARG464
	–	–	–	ARG467
	ARG481	–	–	–
	–	PRO482	–	–
	–	GLY484	–	–
	–	MET485	–	–
	ASP487	–	ASP487	ASP487
	–	–	–	GLU493
Sparfloxacin	–	–	–	PRO230
	–	THR269	–	–
	–	GLY271	–	–
	–	–	GLU291	GLU291
	HIS293	HIS293	–	HIS293
	THR295	–	VAL295	GLN295
	ASP296	–	ASP296	–
	–	SER297	–	–
	–	THR298	–	–
	ASP412	–	ASP412	ASP412
	–	–	MET415	MET415
	–	THR416	–	–
	VAL432	–	VAL432	–
	THR433	–	THR433	–
	GLN434	GLN434	GLN434	GLN434
	LEU451	–	–	–
	–		PRO452	–
	–	–	ASP454	ASP454
	SER457	–	–	–
	GLN460	–	–	GLN460
	ARG464	–	–	–
	ARG467	–	–	ARG467
	ARG481	ARG481	ARG481	ARG481
	–	PRO482	–	–
	GLY484	–	–	–
	MET485	MET485	MET485	–
	ASP487	–	ASP487	ASP487
	–	GLU493	–	GLU493
	–	TYR502	–	–
	–	–	–	ASN556
Pefloxacin	–	–	ASN229	ASN229
	–	–	–	SER231
	–	–	–	GLU291
	HIS293	HIS293	HIS293	HIS293
	THR295	THR295	VAL295	GLN295
	ASP296	ASP296	–	–
	–	SER297	ALA297	–
	–	–	–	THR298
	HIS369	–	–	–
	LYS371	–	–	–
	THR411	–	–	–
	–	ASP412	–	–
	–	–	MET415	MET415
	THR416	–	–	–
	VAL432	–	–	–
	–	GLN434	GLN434	GLN434
	LEU451	–	–	–
	ASP454	–	ASP454	ASP454
	SER457	–	–	–
	GLN460	GLN460	–	GLN460
	–	–	–	ARG464
	ARG481	–	ARG481	–
	–	GLY484	–	–
	MET485	MET485	MET485	–
	ASP487	–	ASP487	ASP487
	–	SER489	–	–
	–	GLU493	–	–
	–	TRP501	–	–
	–	ASP555	–	–

Analysis of the docking scores between NS3 (from different genotype) and different fluroquinolone showed the docking scores for Balofloxacin-NS3 ranged from − 98 to − 111; Ciprofloxacin-NS3 ranged from − 103 to − 114; Enrofloxacin-NS3 ranged from − 121 to − 100; Lomefloxacin-NS3 ranged from − 90 to − 101; Levofloxacin-NS3 ranged from − 90 to − 115; Ofloxacin-NS3 ranged from − 88 to − 106; Sparfloxacin-NS3 ranged from − 87 to − 104; and Pefloxacin-NS3 ranged from − 87 to − 109 (Table 3).

**Table 3:** Molegro docking score for NS3-Fluoroquinolones interactions: Molegro docking score for top 10 poses observed for each fluoroquinolone-NS3 (specific to each genotype) interactions are shown. Previously determined in vitro IC50 (µM) for each fluoroquinolone, except for Levofloxacin (shown as –) is also given.

Ligand (top 10 poses)	IC_50_ (uM)^12^	Genotype 1a (MolDock score)	Genotype 1b (MolDock score)	Genotype 2b (MolDock score)	Genotype 3a (MolDock score)
Balofloxacin	1.37	− 99	− 105	− 112	− 104
Ciprofloxacin	2.99	− 104	− 115	− 108	− 110
Enrofloxacin	1.15	− 101	− 102	− 107	− 122
Levofloxacin	–	− 90	− 116	− 106	− 102
Lomefloxacin	1.14	− 96	− 91	− 102	− 99
Ofloxacin	1.47	− 88	− 97	− 107	− 100
Pefloxacin	4.22	− 103	− 87	− 102	− 110
Sparfloxacin	1.22	− 92	− 88	− 104	− 92

## Discussion

In the current study, we present an in-silico approach to assess the effect of genotype-specific amino acid variations on protein folding and structural architecture and its interaction with the drugs. Our strategy involved computational analysis of NS3 helicase sequence and structures followed by molecular docking of fluoroquinolones on genotype-specific NS3 structures.

In the past era, a substantial number of studies explored the genetic heterogeneity of viral genotypes and its correlation with various aspects of the viral life cycle, including replication, and pathogenesis. Additionally, efforts have been made to unravel inter-genotype clinical and serological variations, functional differences in viral proteins and their effect on host immune system, generation of escape variants and viral epitopes, and on antiviral treatment response^16–20^. For example, the study conducted by Kaneez et al. showed amino acid variability in HCV NS3 1 and 3a and its impact on the structural architecture of the protein. The study also emphasized on the functionally active and conservative residues across selected HCV genotypes. To do so, several bioinformatics-based approaches and computational methods were employed. They reported that genotype-specific variations affected the structural architecture of NS3^21^. Similarly, Ahmed et al*.*^22^ established the impact of sequence heterogeneity within NS5A and core regions on the response to pegylated interferon/ribavirin (PEG‐IFN/RBV) therapy against HCV. The results showed a significant association between heterogeneity in IFN/RBV resistance‐determining region IRRDR of NS5A and SVR, indicating that genetic heterogeneity in IRRDR can potentially serve as a predictor for SVR in HCV-infected patients treated with PEG-IFN/RBV combinatorial therapy^22^.

Another study published by Di Maio et al*.* implemented an in-silico approach to identify NS5B genetic variability in HCV genotypes and its potential effect on the genetic barrier for drug resistance (nucleoside inhibitors (NI)and non-nucleoside inhibitors(NNI))^23^. The impact of these mutations was analyzed via docking of sofosbuvir with the NS5B protein of HCV genotypes. This study suggested that HCV sequence variability in NS5Bcan potentially alter the efficacy of NS5B inhibitors^23^.

The approach we have used here has the advantage that it analyzes the effect of naturally occurring genotype-specific amino acid variations on drug–protein interactions in the absence of drug-induced selection pressure. This method allowed us to correlate genotype-specific sequence heterogeneity with its influence on protein’s structural architecture, and with its effect on variable interactions with selected drugs. This approach can be extended to other viruses to study the clinical significance of genotype-specific sequence variations that influence drug–protein interactions.

In our study, sequence to structure comparison identified the following genotype-specific substitutions: Y241F in genotype 3a (Motif Y), F418Y in genotype 2b (Motif V), and S459T in genotype 1a (Motif VI), whereas V256N in genotype 3a, S294A in genotype 3a, T295V/Q in genotypes 2b and 3a, A297S in genotype 1b, S299T in genotype 1b and 2b, T430V in genotype 2b and 3a, E555D/L in genotype 2b and 3a, P558A/S in genotype 2b and 3a and Q580V/E in genotype 2b and 3a (Figs. 1 and 2).

In the previous structural–functional studies of NS3 HCV, it has been proposed that the residues that play a critical role in NTP and substrate binding are found in the conserved helicase motifs: Motif I/Walker A (207′-GSGKSTK-213′), Ia (223′-YKVLVLNPSVA-233′), Walker B/Motif II (290′-DECH-293′), III (319′-VLATATPP-326′), IV (365′-LIFCHSKKK-373′), V (410′-ATDALMTGYTGDF-422′), and VI (456′*-*VSRSQRRGRTGR-467′)(Fig. 1)^21,24,25^. These helicase signature motifs are oriented in such a manner that their spatial arrangement forms the lining of NTP and substrate binding pocket, located in the cleft between domains 1 and 2^25,26^. Motif Y (Y241) is a characteristic motif of NS3 HCV located between motifs 1a and 1b. Tyrosine241 plays an important role in stacking the adenine base of ADP in correspondence with T419^26^. In addition to these, amino acids at positions 256V/V/V/N, 294S/S/S/A, 295T/T/V/Q, 297A/S/A/A, 299S/T/T/S, 430T/T/V/V, 555E/D/D/L, 558P/P/A/S and 580Q/Q/V/E exhibited genotype-specific variation. These amino acids form the entry side of ssRNA/DNA^21^.

In light of our observations, we speculate that A240S in genotype 3a, A431C in genotype 1a, T433E in genotype 3a, T435I/Y in genotype 2b and 3a, T445S in genotype 3a, E447T in genotype 2b, T449Q/R in genotype 2b and 3a, A500S in genotype 3a and F557L in genotype 1a might be of functional importance. These amino acids are present in the vicinity of the residues involved in the catalytic center and/or substrate binding loop, and therefore, can influence nucleic acid binding and NS3 catalytic activity. Residue Y241 is crucial for forming interactions with the adenine base of ADP^21,26^. In genotype 3a, we observed substitutions at both positions 240 and 241 simultaneously, while the Tyrosine241 was replaced with phenylalanine, alanine at position 240 that was substituted with serine. These substitutions concurrently can affect the binding of ATP molecule and subsequently the helicase activity due to changes in the electrostatic environment of the binding site. In close vicinity of Walker B Motif, lie residues 294 and 295. In this motif, H293 is of prime importance and is implicated in the coupling of ATPase and helicase activity^21^. Amino acid 294 and 295 are not only found in the proximity H293 but are also present at the entry side of the substrate. Therefore, substitutions at these positions, such as S294A and T295V/Q (Figs. 1 and 2), might not only influence entry and binding orientation of ssRNA/DNA but may also affect NTP binding and hydrolysis. Motif V corresponds to the coordination of ATP and substrate binding^21,26,27^. We observed a substitution of amino acid F418Y, adjacent to the functionally important residue 419, involved in NTP binding and catalysis^21^. Here, we speculate little or no effect on the activity because the amino acid change was conservative. Residues 431C/C/A/A, 433T/T/T/E, 435T/T/I/Y, 445T/T/T/S, 447E/E/T/E, 449T/T/Q/R, 500A/A/A/S, 557L/F/F/F may play either a direct or indirect role in ssDNA/RNA interaction and unwinding. This leads us to speculate that genotype-specific variations in these amino acids might influence substrate interaction and helicase activity.

A characteristic structural feature of NS3 HCV is a β-hairpin spanning the amino acids 430–452. This hairpin consists of two conserved phenylalanine residues F438 and F444. Residues V432 and T450 in the hairpin are known to be involved in nucleic acid interactions^28^. V432 intercalates between the DNA bases, stacks the nucleotide bases at 5′ terminal, and locks the enzyme in position, whereas T450 is involved in ssDNA binding to the nucleic acid pocket of the NS3 helicase^28^. F438 and F444 are responsible for the release ssDNA upon ATP binding^28^. It may be suggested that substitutions in the vicinity of these two residues, at positions 430T/T/V/V, 431C/C/A/A, 433T/T/T/E, 435T/T/I/Y, 445T/T/T/S, 447E/E/T/E, 449T/T/Q/R and 451L/V/V/A can alter the flexibility of the loop and subsequently might affect substrate interaction. Q460, a conserved residue of helicase Motif VI, plays a key role in ATP binding and coupling ATP hydrolysis with helicase translocation^26^. W501 is a critical residue for nucleic acid binding^26^.

To gain insights in drug–protein binding pattern across the HCV genotypes; molecular docking simulations were performed with NS3 protein models of each candidate genotype against a batch of 8 fluoroquinolones (Sparfloxacin, Ciprofloxacin, Balofloxacin, Levofloxacin, Lomefloxacin, Enrofloxacin, Pefloxacin, and Ofloxacin) previously shown to exhibit inhibitory potential against HCV NS3 helicase under in vitro conditions^12^. In our analysis, it was observed that the fluoroquinolones interacted with amino acids that constitute the helicase catalytic core via H-bond as well as non-bond interactions (Supplementary Table 2). Several studies have demonstrated that both H-bonds and hydrophobic interactions stabilize the ligands at the target site and assist in altering binding affinity and drug efficacy^29^. Furthermore, biological activity of the drug increased with increase in the number of hydrophobic interactions in the core of drug–target interface^29^. The residues that are functionally important and were found involved in H-bond as well as non-bond interactions with our drugs are P230, S231, T269, K371, R393, W501 and Y502^25,26^ (Figs. 5 and 6; Table 2; Supplementary Table 2). Residues S231, T269, W501, and Y502 are functionally important as they are known to facilitate the interactions of NS3 with a 3′ segment of substrate/ssDNA. P230 and S231 are key residues involved in DNA binding. S231 interacts with substrate/ssDNA via bridging water molecule, while T269 is thought to be indispensable for helicase activity of NS3. K371 and R393, crucial residues of NS3 active site, are involved in substrate binding, where R393 makes interactions with the 5′ segment of ssDNA and coordinates in translocation of NS3 helicase^25,26^; in addition to this, K371 participate in stabilizing the interaction with ssDNA/RNA^30^. W501 stacks the nucleotide bases near the 3′ end of the substrate/ssDNA and acts as a bookend along with V432 that defines a central binding cavity^26^.

Fluoroquinolones, Sparfloxacin (in genotype 3a), Balofloxacin (in genotype 2b and 3a), and Lomefloxacin (in genotype 3a) targeted residue P230 (Fig. 5 and Table 2) that might hinder in substrate binding. Enrofloxacin, Levofloxacin, and Pefloxacin (in genotype 3a) were found to interact with residue S231. Interactions of the Enrofloxacin, Levofloxacin, and Pefloxacin with these residues might affect ATP binding and transition as well as enzyme–substrate interaction. Likewise, Sparfloxacin (in genotype 1b) targeted T269 (Table 2) that can potentially inhibit substrate binding and subsequently NS3 unwinding activity. R393 is thought to be a key residue that makes direct contact with ssDNA/RNA as well as facilitates nucleic acid unwinding^28^. Therefore, drugs such as Balofloxacin (in genotype 1a and 3a), Ciprofloxacin (in genotype 3a) targeting residue R393 (Fig. 5 and Table 2) can greatly affect substrate binding, NS3 translocation and subsequently nucleic acid unwinding.

W501 is a critical amino acid of substrate binding cleft of NS3 HCV that serves as a bookend residue; stacking nucleotide of ssDNA/RNA at 3′ terminal preventing the protein from sliding along the nucleic acid and facilitates helicase translocation^26,28^. Ciprofloxacin, Pefloxacin and Enrofloxacin (in genotype 1b) and Levofloxacin and Lomefloxacin (in genotype 3a) interacted with W501, while most fluoroquinolones with interacted residues in their proximity of W501, namely E493 (present in genotypes 1b, 2b, and 3a) (Fig. 5 and Table 2), which might cause hindrance in nucleic acid interaction and can potentially inhibit the helicase activity of NS3. Maga et al. identified a series of compounds out of which QU663 exhibited a strong binding affinity with NS3. QU663 binds to the RNA binding cleft and makes direct contact with residues R393 and W501^31^ that are crucial for substrate binding and helicase translocation. The present study showed that fluoroquinolones we tested also targeted the same crucial residues in the substrate-binding catalytic cleft, namely, W501 and R393 (Fig. 5).

We identify a few limitations of our study. A major limitation of this study was that our analysis was performed entirely in silico. We used two crystal structures (for 1a and 1b) and used a homology modeling approach to construct NS3 structures for genotypes 2b and 3a. Homology modeling heavily relies on the identification of the correct template, selected based on sequence identity^32^. A sequence identity above 50% generally tends to generate reliable structures with limited errors in loops and side-chain positing, while structures below 30% can have serious folding errors^32^. Additionally, regions in the query sequence that share low sequence similarity with the template protein, even though the rest of the protein show a high sequence similarity, can lead to erroneous folding. We tried reducing this bias/error by using three different homology modeling programs, validating and checking constructed structures for various errors and using a template that exhibited excellent similarity with our query sequences. Interestingly, all three programs identified 1A1V as a suitable template sharing more than 90% similarly with the sequence, and all three programs gave the same structures. Additionally, our structures passed the 3D verification (performed using Verify 3D software) as at least 80% of the amino acids have scored >  = 0.2 in the 3D/1D profile. Similarly, the structures were valid on the Ramachandran plot as most of the amino acids were under the permissible regions. The other limitation we anticipate is with the molecular docking approach we used. In absence of experimentally characterized binding sites and experimentally derived binding energies, molecular docking analyses can be unreliable and can potentially miss out several key residues, present artificial interaction with residues and/or under- or over-estimate the binding scores^33^. We tried to address this issue by validating our docking approach using two previously reported structures bound to the ligand. We adopted a ‘blind docking’ approach, where the binding site of the ligand was not defined. Our software predicted the same pose for the ligand and also identified all the residues previously reported for the two structures. Another limitation that we identify is use of docking score to rank the poses and selection the top pose with highest docking score. Docking programs produce one (or several) different poses for every ligand, and further rank different compounds based on their scoring functions^34^. Several studies suggest that binding energies/scores predicted by the docking might be incorrect, despite the correctly predicted binding pose^34^. These can be overcome by using more robust approached such as MD simulations followed by WaterMap analysis; however, these approaches can be time-consuming and computationally demanding^34^. Nonetheless, as the experimentally solved structures continue to grow, the boundaries that differentiate between reliable and unreliable predictions will narrow and the capacity of the docking tools to predict correct poses/interactions will increase^32^. However, to gain further insights into the mechanism, site-directed mutagenesis of crucial residues followed by functional assays may be employed. These analyses should be supplemented by observations from the crystal structures of drug–enzyme complexes.

In conclusion, our approach provides an in-depth analysis of sequence variation in HCV NS3 protein across the selected genotypes and identifies active site amino acid residues crucial for drug–protein interactions. This approach can help study inter-genotype sequence heterogeneity in viral proteins and its correlation with the response to the antiviral treatment regimen.

## Methods

### Retrieval of HCV NS3 sequences and sequence analysis

A total of 1703 HCV NS3 sequences, belonging to genotype 1a, 1b, 2b, and 3a were downloaded in FASTA format from Los Alamos HCV Database (https://hcv.lanl.gov/content/index)^35^. Other genotypes were not considered because their sequences were not present in the database in sufficient numbers to conduct a meaningful analysis. Out of these sequences, 687, 667, 101 and 248, belonged to, respectively, genotype 1a, 1b, 2b, and 3a. Sequences for each genotype were aligned separately and edited using the MEGA 6.0 software, implementing the ClustalW algorithm. The aligned nucleotide sequences were then used to generate consensus sequences for each of the candidate genotypes using the advanced ‘Consensus maker’ tool with 0.7 thresholds (i.e. residues represented in ≥ 70% of the sequences were used to generate a consensus) (http://www.hiv.lanl.gov/content/sequence/CONSENSUS/consensus.html). In all genotypes, almost all consensus residues/sites were present in 99% of the sequences, with exception of a few sites, where consensus residues were present in 70–85% sequences (Supplementary Table 1). The consensus sequences of NS3 for genotypes 1a, 1b, 2b, and 3a were subsequently translated to amino acid sequences using the ExPASy translate tool^36^. Additionally, reference sequences for genotypes 1a, 1b, 2b, and 3a were downloaded in FASTA format PDB (genotype 1a: PDBid:1A1V and genotype 1b: PDBid:1CU1) and UniProt (genotype 2b: UniProt ID Q9DHD6 and genotype 3a: UniProt ID Q81495) databases. For genotype variation analysis, the genotype sequences were aligned with each other using the ClustalW algorithm implemented in MEGA 7 software^37^. Additionally, each genotype consensus sequence was also aligned, using MEGA 7, with its reference sequence to compare the difference in consensus sequences and the reference sequences.

Inter-subtype sequence similarities and differences were determined by constructing a sequence identity matrix using ‘Bioedit’ software^38^. Two ID matrices were constructed: one with a full-length sequence of HCV NS3 helicase comprising 623 amino acids, and the other comprising of amino acids spanning Pro230-Cys584 (termed in this study as fluoroquinolone binding region). This region was selected because fluoroquinolones are found to form interactions with different amino acids within this region^39^. Additionally, this region also contains the previously reported helicase active site, spanning from Arginine393 to Tryptophan501^8,40,41^.

### Retrieval of HCV helicase structures and protein homology modeling

PDB and UniProt databases were searched for the available genotype-specific NS3 helicase structures and crystal structures were only found for genotypes 1a and 1b. These structures (genotype 1a: PDBid:1A1V and genotype 1b: PDBid:1CU1) were downloaded on *.pdb* format. For genotypes 2b and 3a, the Homology Modeling approach was adopted for the generation of 3D protein structures^10,42^. For accuracy and reliability, 3D models of NS3 were generated using three different homology modeling tools, namely the CPH model, the Swiss model and Phyre 2^43–45^. The structures were visually inspected in Discovery Studio Visualizer version 4.0 (DSV4.0; *Dassault Systèmes BIOVIA, Discovery Studio Visualizer, version 4.0, San Diego: Dassault Systèmes, 2016;* Retrieved from http://accelrys.com/products/discovery-studio/), and thereafter saved in PDB format. Subsequently, the structures were verified using the Verify 3D tool, while the energy minimization and validation were performed using the GROMACS tool^46^ and Ramachandran plot analysis implemented in DSV4.0.

### Fluoroquinolone structures

In this study, we used a panel of 8 fluoroquinolones, namely Sparfloxacin, Ofloxacin, Balofloxacin, Pefloxacin, Levofloxacin, Lomefloxacin, Ciprofloxacin, and Enrofloxacin, that have previously been reported to effectively inhibit HCV NS3 helicase^12^. Structures of these drugs were retrieved from PubChem Database in 3D SDF format^47^. Before docking analysis, SDF structures were converted to PDB format using DSV4.0.

### Analysis of genotype-specific fluoroquinolone-NS3 interactions

Molecular docking simulations were performed to analyze the genotype-specific protein–ligand interactions. The docking approach was validated by performing blind docking (assuming drug binding site to be anywhere on the protein) on two previously reported complexes of NS3 bound to inhibitors, one a natural analog M2^48^ and other inhibitor ITMN-3479 (http://www.rcsb.org/pdb/explore/litView.do?structureId=3RVB). Molecular docking studies and conformational analysis were conducted by Molegro Virtual Docker^49^ and DSV4.0 software. Molegro Virtual Docker binding site was set to default, where the Molegro identifies the binding ligand binding sites in the protein. For our analysis, Molegro identified two binding sites, one located in the DNA binding domain, which contained the key residues within the active site of NS3, as identified by previous reports^8,40,41^, and others in the ATP domain of the helicase. The 8 fluoroquinolones were docked individually on each of the four genotype-specific models of NS3, into the identified cartesian space. Molecular docking was performed using standard precision protocols with default parameters of Molegro Virtual Docker. A total of 2000 interactions were simulated, and out of those top 10 poses was selected based on docking energies. Using these poses, analysis of genotype-specific variations on NS3-fluoroquinolone binding was carried out. Visualization of docking poses and analysis of drug–protein interactions were performed using DSV4.0.

## Supplementary information

Supplementary Information.
