# Supplementary material for: An in silico approach to analyze HCV genotype-specific binding-site variation and its effect on drug–protein interaction
Source: Sci Rep. 2020 Nov 30;10:20885. doi: 10.1038/s41598-020-77720-9 (PMC7705671; doi:10.1038/s41598-020-77720-9)
Supplement: Supplementary file 1 — Supplementary Information. [file 41598_2020_77720_MOESM1_ESM.pdf]

Title:

An *in silico* approach to analyze HCV genotype-specific binding-site variation and its effect on drug-protein interaction

Authors and Affiliations:

Ramsha Khalid[1], Muhammad Faraz Anwar[2], Muhammad Aanish Raees[3], Sadaf Naeem[1], Syed Hani Abidi[2,*,+], Syed Ali[4,*,+]

[1] Department of Biochemistry, University of Karachi, Karachi, Pakistan

[2]Department of Biological and Biomedical Sciences, Aga Khan University, Karachi, Pakistan

[3]Department of Cardiac Surgery, Vanderbilt University, US

[4]Nazarbayev University School of Medicine, Nazarbayev University, Kazakhstan

+ These authors have contributed equally to this work

***Corresponding Authors:**

Syed Hani Abidi
Department of Biological and Biomedical Sciences, Aga Khan University, Karachi, Pakistan
Email: m.haniabidi@gmail.com

Syed Ali
Department of Biological Sciences, Nazarbayev University School of Medicine, Nazarbayev University, Astana, Kazakhstan
Email: syed.ali@nu.edu.kz

Supplementary Table 1: HCV genotype amino acid analysis: For each genotype, key and minor amino acid at each position, and their percentage in sequence alignment are given.

Position (Genotype 1A)	190	191	192	193	194	195	196	197	198
AMINO ACID OCCURRENCE (%)	99.9 P	99.9 P	97.8 A	99.9 V	99.9 P	96.05Q	97.9 S	84.85 F	99.9Q
	0.05 S		2.15 V			3.9 E	2 T	15.1Y	
							0.05C		
							0.05I		

Position (Genotype 1B)	190	191	192	193	194	195	196	197	198
AMINO ACID OCCURRENCE (%)	97.3 P	99.2 P	95.3 A	98.8 V	99.7 P	99Q	94.7 T	95.6 F	98.8 Q
	0.1 A	0.05 L	0.05 L	0.05 G	0.1S	0.05 A	0.1V	0.05 I	0.1E
		0.05 R	0.25T	0.05 Y	0.05 T	0.05 E	0.05 D	0.15 S	0.05 H
			1.15		0.05 R	0.05 L	0.05 H	1.8Y	0.05 K
						0.05 R	1A		
						0.05 H	3.05 S		

Position (Genotype 2B)	190	191	192	193	194	195	196	197	198
AMINO ACID OCCURRENCE (%)	100 P	100 P	95.8 A	100 V	100 P	100 Q	70.4 S	89.1 Y	100 Q
			0.84T				0.84A	10.92F	
			3.36V				5.04S		
							3.36H		
							23.69 T		

Position (Genotype 3A)	190	191	192	193	194	195	196	197	198
AMINO ACID OCCURRENCE (%)	99.5 P	100P	97.2 A	100V	100P	99.2 Q	97.4 S	99.7 Y	99.5 Q
			2.55 V			0.765N	0.255G	0.255C	
							0.255N		
							2.04T		

199	200	201	202	203	204	205	206	207	208	209	210	211
99.9 V	99.85A	99.9 H	99.9L	99.9H	99.9A	99.9P	99.9T	99.9G	99.9S	99.9G	99.9K	99.9S
	0.1V	0.05 Y										

199	200	201	202	203	204	205	206	207	208	209	210	211
99.3 V	98.6A	97.9 H	97.0 L	98.5 H	97.4 A	97.2 P	97.8 T	98.2 G	99.3 S	98.3 G	98 K	99.5 S
0.05 W	0.1G	0.05 I	0.05 C	0.05 T	0.05 L	0.05 L	0.05 L	0.05 A	0.05 A	0.05 A	0.05 R	0.05 A
0.15 I	0.05 H		0.05 I	0.05 Q		0.05 A		0.05 R				
	0.05 V											
	0.2S											

199	200	201	202	203	204	205	206	207	208	209	210	211
100 V	100 G	100 Y	100 L	100 H	100 A	100 P	96.6T	100 G	100 S	99.2	100 K	100 S
										0.84R		

199	200	201	202	203	204	205	206	207	208	209	210	211
99.5 V	99.5 G	100Y	99.2 L	100H	99.5 A	100P	100T	99.5 G	100S	100G	100K	100S
			0.255F		0.255T			0.255D				
					0.255V							

212	213	214	215	216	217	218	219	220	221	222	223	224
99.9 T	99.9 K	99.9V	99.9 P	99.9A	99.9A	99.9Y	99.9 A	99.9A	99.85Q	99.9 G	99.9Y	98.25K
0.05 S	0.05R		0.05L				0.05 T		0.05 K	0.05S	0.05S	0.05E
									0.05 R			0.65N
												0.65T
												0.35Q

212	213	214	215	216	217	218	219	220	221	222	223	224
96.5 T	90.6 K	99.4 V	98.3 P	98.8 A	95.9 A	96.3 Y	98 A	96 A	99.1 Q	99.2 G	97.5 Y	98K
0.1N		0.05 A	0.05 R	0.1S	0.1E	0.05 M	0.05 Q	0.15 D	0.05 K	0.05 R	0.05 T	0.1Q
0.05 L		0.05 C		0.01V	0.05 R		0.05 A	0.9 G			0.1F	0.1S
				0.05 L	0.05 G		0.05 V	0.15P				0.05 M
				0.05 V				1.2 S				0.15 N
								0.3 T				0.15 R

212	213	214	215	216	217	218	219	220	221	222	223	224
100 T	100 K	100 V	98.3 P	95.0 A	96.6A	100 Y	99.2 A	97.5S	100 Q	100 G	99.2 Y	99.2 K
				1.68V			0.84T	1.68A			0.84F	0.84R
								0.84I				

212	213	214	215	216	217	218	219	220	221	222	223	224
99.7 T	99.2 K	99.7 V	100P	99.5 A	99.7 A	99.5 Y	99.5 V	99.2 A	99 Q	100G	99.7 Y	79.6 N
0.255A	0.51R	0.255I		0.51T	0.255T		0.255A	0.255T				0.255K
								0.51V				5.61D
												1.02H
												2.55S
												40.765T

225	226	227	228	229	230	231	232	233	234	235	236	237
99.9V	99.9L	99.9V	99.9 L	99.9N	99.9P	99.9S	99.9 V	99.9A	99.9A	99.9 T	99.85L	99.7G
			0.05I				0.05I			0.05S	0.05M	0.1A
											0.05V	0.15S

225	226	227	228	229	230	231	232	233	234	235	236	237
96.4 V	95.7 L	98.8 V	97.5 L	99.3 N	97.4 P	97.6 S	97.5 V	98.8 A	98.4 A	98.1 T	95.5 L	87.2 G
0.05 C	0.1R	0.05 I		0.05 T	0.05 R	0.05 F	0.05 L	0.1T	0.05 T	0.05 P	0.05 T	0.05 A
0.05 I	0.05 S	0.05 S				0.05 P		0.05 P	0.05 P			0.05 V
						0.1T						0.2N
												8.6 S

225	226	227	228	229	230	231	232	233	234	235	236	237
100 V	100 L	98.3 V	100 L	100 N	100 P	100 S	100 V	100 A	100 A	100 T	99.2 L	100 G
		1.68G									0.84R	

225	226	227	228	229	230	231	232	233	234	235	236	237
99.5 V	99.7 L	99.7 V	98.5 L	100N	100P	99.7 S	100V	99.7 A	99.5 A	100T	99 L	99.7 G
	0.255H	0.255M	0.51M					0.255T	0.255T		0.255I	
			0.51P						0.255V			

238	239	240	241	242	243	244	245	246	247	248	249
99.9F	99.9G	99.3 A	99.9Y	99.75 M	99.9S	99.35 K	99.9A	99.05H	99.9 G	74 V	99.15 D
		0.65V		0.05I		0.6R		0.9Y	0.05E	26.95I	0.75E
				0.15V							0.05 G

238	239	240	241	242	243	244	245	246	247	248	249
97.0 F	98 G	96.2 A	96.9 Y	99.7 M	95.6 S	97.6 K	99.4 A	84.2 H	98.2 G	70 V	95.1 D
0.1L	0.1E	0.1M	0.1F	0.05 C	0.1P	0.1T	0.1S	0.05 F	0.1V	0.1F	0.05 T
0.05 Y		0.1R	0.05 I	0.05 F	0.05 Q	0.05 A	0.05 H	0.05 L	0.05 Q	0.05 G	3.8E
		0.05 G	0.05 T	0.2V	0.3 A	0.05 K	0.05 M	0.05 T		0.05 L	0.3 N
		0.35P			0.15T	0.8R		13.5Y		23.75I	
		0.2S								0.1M	

238	239	240	241	242	243	244	245	246	247	248	249
100 F	100 G	100 A	100 Y	98.3 M	100 S	96.6K	100 A	94.1H	100 G	95.8 I	98.3 N
				1.68L		2.52R		5.88Y		4.2V	1.68D
						0.84T					

238	239	240	241	242	243	244	245	246	247	248	249
100F	99 G	90.8 S	76 F	100M	98.5 S	95.7 R	100A	96.4Y	99.7 G	99.5 I	99.7 D
	0.51S	0.255N	0.255S		0.51P	0.51S		0.255D	0.255V	0.255T	0.255E
	0.51D	1.02A	23.7Y		1.02A	1.53H		3.06H		0.255V	
		7.9T				2.29K					

250	251	252	253	254	255	256	257	258	259	260	261
98.85P	99.9 N	99.85I	99.9R	99.9 T	99.9G	98.8 V	99.9 R	99.9 T	99.85I	99.9T	99.9 T
1.05A	0.05S	0.1T		0.05K		1.15 A	0.05K	0.05P	0.1V		0.05A
0.05T											

250	251	252	253	254	255	256	257	258	259	260	261
97.0 P	87.7 N	94.3 I	98.8 R	97.0 T	99.5 G	91.6V	97.9 R	98.4 T	95.9 I	98.1 T	97.0.1 T
0.05 L	0.05 T	0.05 M	0.05 E	0.05 L	0.05 W	1.38A	0.05 K	0.05 P	0.05 L	0.1A	0.05 R
	1.5 G	2.05 L	0.05 K	0.55S		4.4I				0.05 P	
	8.75 S	0.05S				0.11M					
	0.25C	0.25T				0.69T					
		0.65 V									

250	251	252	253	254	255	256	257	258	259	260	261
100 P	99.2 N	99.2 I	95.0 R	100 T	100 G	98.3 V	100 R	100 T	99.2 V	98.3 T	100 T
	0.84H	0.84M	0.84G			0.84A				1.68M	
			0.84T			0.84M					

250	251	252	253	254	255	256	257	258	259	260	261
100P	100N	92.9 I	99.5 R	100T	100G	95.9 N	100R	99.5 T	94.9 V	99.7 T	100T
		7.14V	0.255C			1.75D		0.255A	0.765A	0.255A	
			0.255H			1.75T			11.75I		
						0.51S					

262	263	264	265	266	267	268	269	270	271	272	273	274
98.85 G,	98.2S	99.9 P	99.85I	99.9T	99.9Y	99.9S	99.9T	99.9 Y	99.9G	99.9K	99.9 F	99.9 L
1.1 S	1.75 A	0.05S	0.1V					0.05H			0.05L	0.05F

262	263	264	265	266	267	268	269	270	271	272	273	274
94.7 G	91.0 A	71.54P	96.51I	97. T	97.0 Y	99.4 S	98.8 T	95.8 Y	98.4 G	98 K	95.9 F	97.5 L
0.05 A	0.1D	0.98A	0.07L	0.05 R	0.1H	0.05 P	0.1P	0.05 H	0.1C	0.1S	0.1S	0.1F
0.05 N		0.04G	1.24V		0.05 C			0.05 M	0.05 A	0.05 N	0.05 L	0.05 V
		0.07L							0.05 V			
		25.23S										

262	263	264	265	266	267	268	269	270	271	272	273	274
100 G	98.3 D	72.9 P	100 I	100 T	100 Y	100 S	99.2 T	100 Y	98.3 G	100 K	100 F	73.8 L
	1.68E	1.68A					0.84I		1.68A			21.17 I
		23.69 S										5.04 V

262	263	264	265	266	267	268	269	270	271	272	273	274
100G	99.5 A	91.1 K	99.5 L	100T	99.5 Y	100S	99.5 T	100Y	100G	100K	99.7 F	99.5 L
	0.51S	0.255A	0.51P		0.51N		0.51 A					0.255P
		8.67R										

275	276	277	278	279	280	281	282	283	284	285	286	287
99.9 A	99.9D	99.9 G	99.9G	99.9C	99.9 S	99.9G	99.9G	99.85A	99.9 Y	99.9 D	99.9I	99.9I
0.05V		0.05A			0.05T			0.1V		0.05G		

275	276	277	278	279	280	281	282	283	284	285	286	287
98 A	97.8 D	97.6 G	97.7 G	97.4 C	95.7 S	98.4 G	97.7 G	98.5 A	97.0 Y	99.4 D	98.8 I	99.6 I
0.1P	0.1T	0.1V	0.1A	0.1A	0.1L	0.1A	0.1A	0.1P	0.05 C	0.1T	0.1S	
0.05 P	0.05 K	0.05 N			0.05 H	0.05 R		0.05 C	0.05 M			
	0.05 N	0.05 S							0.05 T			

275	276	277	278	279	280	281	282	283	284	285	286	287
100 A	98.3 D	98.3 G	100 G	100 C	88.2 S	92.4A	100 G	100 A	100 Y	100 D	70.6 V	100 I
	1.68N	1.68E			9.24A	1.68G					29.411 I	
					0.84P	0.84P						
					1.68Q	1.68S						
					10.84A							

275	276	277	278	279	280	281	282	283	284	285	286	287
100A	99.5 D	100G	99.7 G	99.7 C	100S	99.7 G	100G	99.7 A	100Y	99.7 D	99 V	99.2 I
	0.51G			0.255Y		0.255A		0.255V		0.255G	0.51 A	0.255S
											0.51I	0.51V

288	289	290	291	292	293	294	295	296	297	298	299	300
99.9I	99.9C	99.9D	99.9E	99.9C	99.9H	99.9S	99.9T	99.9D	98.2A,	99.9T	98.8 S,	99.75 I,
									1.75 S		1.15 T	0.2 V

288	289	290	291	292	293	294	295	296	297	298	299	300
89.71I	98.5 C	99.7 D	99 E	99.1 C	98.3 H	97.9 S	96T	98.9 D	89.28S	97.9 T	78.92S	94.2 I
0.04L	0.1V	0.1M	0.1S	0.1A	0.1R	0.1Q	0.04C	0.1T	9.01A	0.1L	22.97T	0.1S
8.4M				0.05 Y	0.05 I	0.05 P	1.85I		0.04P	0.05 P		
0.33V					0.05 T		1.42V		0.07W			

288	289	290	291	292	293	294	295	296	297	298	299	300
99.2 I	100 C	96.6D	100 E	100 C	100 H	71.4S	100 V	96.6D	93.3 A	100 T	97.5T	96.6I
0.84M						28.57 A			6.7S		2.52 S	

288	289	290	291	292	293	294	295	296	297	298	299	300
99.7 I	100C	100D	99.2 E	99.7 C	100H	99.7 A	100Q	99.5 D	99.7 A	100T	98S	99.7 I
0.255T			0.51D	0.255Y		0.255S		0.51G	0.255V		0.51G	0.255V
											1.53 T	

301	302	303	304	305	306	307	308	309	310	311	312	313
99.9 L	99.9G	99.9I	99.9 G	99.9T	95.85 V	99.9L	99.9D	99.9Q	97.5 A	99.9E	99.9T	99.9 A
0.05S					3.85 A				2.45S			0.05E
					0.05D							
					0.2 T							

301	302	303	304	305	306	307	308	309	310	311	312	313
96.5 L	98.7 G	88.7 I	98.4 G	97.1 T	97.7 V	99.0 L	98.5 D	99.3 Q	99.0 A	99.0 E	99.4 T	99.2 A
	0.05 S	0.1S	0.05 A	0.05 L	0.1S	0.1W	0.1E	0.1K	0.1R	0.1R	0.1R	0.1L
			0.05 D	0.05 Q	0.05 F		0.05 I			0.05 K	0.1M	0.05 C
			0.05 V	0.05 S			0.05 T					

301	302	303	304	305	306	307	308	309	310	311	312	313
100 L	100 G	100 I	100 G	100 T	100 V	100 L	100 D	100 Q	100 A	100 E	99.2 T	100 A
											0.84S	

301	302	303	304	305	306	307	308	309	310	311	312	313
100L	100G	100I	100G	99.7 T	98.5 V	100L	99.7 D	99.5 Q	99.7 A	99.7 E	99 T	98.2 A
					1.53A		0.255G	0.51R	0.255T	0.255G	0.51A	1.53T
											0.51S	

314	315	316	317	318	319	320	321	322	323	324	325
99.9G	98.8 A	99.9R	83.8 L	84.9 V	98.65 V	99.9L	99.9A	99.9T	99.9A	99.9 T	99.9P
	2.2V		16.15 M	15.05T	1.25I					0.05S	
	0.05T				0.05M						

314	315	316	317	318	319	320	321	322	323	324	325
99.9 G	97.1 A	96 R	95.9 L	97.7 V	98.6V	99.7 L	99.6 A	99.4 T	98.5 A	96.5 T	98.4 P
0.1E	0.1R	0.1G	0.05 F	0.1S	0.1C	0.1S	0.1P	0.1P	0.05 L	0.05 H	0.1L
			0.05 I	0.05 C	0.05 A				0.05 Q	0.05 R	
			0.05 S		0.05 I				0.05 T		

314	315	316	317	318	319	320	321	322	323	324	325
96.6G	71.4A	98.3 R	100 L	98.3 V	100 V	100 L	100 A	100 T	99.2 A	100 T	100 P
	28.5V	1.68K		1.68T							

314	315	316	317	318	319	320	321	322	323	324	325
99.2 G	99.7 V	99.7 R	99.7 L	99 T	99.7 V	99.7 L	99.5 A	100T	99.2 A	99.7 T	99.2 P
0.765R	0.255A	0.255G	0.255P	0.765V	0.255I	0.255F	0.255T		0.51R		0.765S
				0.255A			0.255V				

326	327	328	329	330	331	332	333	334	335	336	337	338
99.9P	99.9G	99.9S	83.35 V	99.85 T	99.9V	99.8 P	99.9H	99.9 P	99.9N	99.9I	95.4E	99.9E
			16.6I	0.05A		0.05R		0.05S			4.55Q	
				0.05I		0.1S						

326	327	328	329	330	331	332	333	334	335	336	337	338
98.4 P	98.8 G	97.8 S	0.954 V	98.8 T	98.8 V	97.8 P	97H	88.48P	93.8 N	97.3 I	97.6E	99.0 E
0.1R	0.1D	0.1R	0.1L	0.1P	0.1C	0.1H	0.05 I	0.04Q	0.1H	0.1S	1.49Q	0.05 K
			0.05 F		0.05 A	0.05 A	0.05 T	7.34S	0.1I		0.33D	0.05 R
					0.05 F	0.1 S		0.18T	0.05 C			
					0.1 T			2.22A				
								0.76H				

326	327	328	329	330	331	332	333	334	335	336	337	338
100 P	100 G	94.1T	100 V	100 T	100 T	100 P	100 H	92.4S	97.5N	100 I	99.2 E	99.2 E
		5.88S						4.2A	2.52D		0.84D	0.84G
								0.84N				
								1.68P				
								0.84T				

326	327	328	329	330	331	332	333	334	335	336	337	338
100P	99.2 G	100S	99.5 I	99.5 T	99.7 V	98.7 P	99.7 H	93.9 S	99.2 N	100I	100E	99.7 E
	0.255C		0.51V	0.51A	0.255A	0.51 L	0.255L	0.255A	0.51 H			0.255G
						0.51S		5.61P				

339	340	341	342	343	344	345	346	347	348	349	350	351
95 V	99.9 A	99.9L	99.9 S	98.1 T	98.1 T	99.9G	99.9E	99.9 I	99.9P	99.9F	99.9Y	99.9G
4.95 A	0.05T		0.05P	0.05I	0.65Q			0.05V				
				1.7 N	1.15I							
				0.1S	0.05A							

339	340	341	342	343	344	345	346	347	348	349	350	351
96.29V	95.09A	97.5 L	95.8 S	90.7 N	70.03T	98.5 G	97.9 E	93.6 I	98.7 P	97.9 F	97.9 Y	98.4 G
0.51A	3.38G	0.1C		0.05 I	3.49V	0.1E	0.1R	0.1S	0.15S	0.1L	0.1M	0.15A
1.96I	0.04P			0.05 Q	0.4A			0.05 I		0.15S	0.05 K	
0.22M	0.04T				0.87D			4.7 V				
0.22S					0.04G							
0.07T					15.85I							

339	340	341	342	343	344	345	346	347	348	349	350	351
100 V	98.3 A	100 L	99.2	95.8 H	99.2 E	100 G	99.2 E	99.2 I	100 P	100 F	100 Y	100 G
	1.68G		0.84S	0.84C	0.84D		0.84Q	0.84V				
				1.68Q								
				1.68R								

339	340	341	342	343	344	345	346	347	348	349	350	351
99.7 V	99.7 A	99.7 L	97.7 G	93.9 S	99.2 E	100G	99.7 E	99.2 I	99.7 P	100F	100Y	100G
0.255M	0.255 V	0.255P	2.29S	0.255L	0.765D		0.255G	0.765V	0.255S			
				5.35P								
				0.255T								

352	353	354	355	356	357	358	359	360	361	362	363	364
99.9K	99.9 A	99.9I	99.9P	98.2L	99.9 E	87.8 V	99.85I	1990 K,	99.9G	99.9G	99.9R	99.85H
	0.05T			1.75I	0.05G	10.95 A	0.05T	00.45 R				0.05N
						0.05I	0.05V					0.05Y
						01.15T						

352	353	354	355	356	357	358	359	360	361	362	363	364
97.8 K	98.9 A	97.9 I	97.7 P	81.3 I	0.95E	79.8 T	98.5 I	93.3 K	97.0 G	98.3 G	97.8 R	96.6 H
0.7R	0.1P	0.1F	0.05 H	0.1T	0.1R	0.1P	0.1V	0.05 E	1.9 E	0.05 A	0.05G	0.1G
	0.05 G	0.1S	0.65 S	0.05 M	0.1S	0.1F	0.1S	0.05 G				0.05 R
	0.05 T	0.05 V		0.05 S	0.6A	20.9A	0.05 T	0.05 Q				0.05 Y
	0.05 V	0.05 T		14.25L	1.45D	1.3L		0.05 T				
	0.3 S	0.4L		0.6V	0.2Q	0.2M		3.5 R				

352	353	354	355	356	357	358	359	360	361	362	363	364
98.3 K	99.2 A	99.2 I	100 P	100 L	94.1A	77.8 F	99.2 I	96.6K	100 G	100 G	100 R	100 H
1.68R	0.84D	0.84L			2.52 S	1.68C	0.84V	0.84G				
					3.36T	5.88H		2.52 R				
						1.68L						
						0.84N						

352	353	354	355	356	357	358	359	360	361	362	363	364
96.7 K	99.5 A	98.2 I	100P	74.5I	98.5 A	70.9 L	94.4 L	98.5 K	100G	99.7 G	100R	99 H
3.31R	0.51T	1.75L		15.5L	0.255V	0.51 C	5.35 I	0.255E		0.255R		0.51R
				8.9M	1.27 D	0.51F	0.255V	1.02R				0.51Y
				0.51T		1.02S		0.255T				
				0.51V		20.08Q						

365	366	367	368	369	370	371	372	373	374	375	376
99.9 L	1996 I	99.9F	99.9C	99.9H	99.9S	89.7 K	83.9 K, 16.1 R	99.9K	99.9C	99.9D	99.9E
0.05P	0.15 V					5.1 R					

365	366	367	368	369	370	371	372	373	374	375	376
98.8 L	96.5 I	98.8 F	97.5 C	98.4 H	98.8 S	90.6 K	96.6 K	97.7 K	99.0 C	96 D	98.7 E
0.05 I	0.1S		0.1S	0.1A	0.1I	0.1P	0.05 S	0.15 R	0.05 Y	0.1N	0.1T
0.05 P	0.05 T		0.1W		0.05 T	0.05 K	0.05 A		0.05 T	0.05 N	0.2D
0.05 T	0.05 V		0.05 Y			6.75 R	1.75 R		0.05 N	0.05 V	
										0.1 A	
										0.15 E	

365	366	367	368	369	370	371	372	373	374	375	376
100 L	98.3 I	100 F	100 C	100 H	100 S	95.8 K	98.3 K	99.2 K	100 C	100 D	100 E
	1.68V					4.2R		0.84R			

365	366	367	368	369	370	371	372	373	374	375	376
99.5 L	93.6 I	99.7 F	100C	99.7 H	100S	93.4 K	99.7 K	100K	100C	86.7 D	99.5 E
0.51P	6.377V			0.255R		6.377R	0.255N			13.26E	0.51K
						0.255M					

377	378	379	380	381	382	383	384	385	386	387	388
99.9L	99.9 A	97.8 A	99.85K	99.9L	95.6 V	99A	89.9 L	99.9 G	81.6 I, ,	99.85N	99.9 A
	0.05T	1.1G	0.1R		0.1A	0.85 G	10.05M	0.05S	0.05A	0.05H	0.05V
		0.45S			0.05F	0.05T			1.75L	0.05S	
		0.6 T			0.15 I	0.05V			16.55V		
					1.75S						

377	378	379	380	381	382	383	384	385	386	387	388
96.9 L	97.5 A	88.8 A	92.05 K	97.1 L	89.1 S	70.5 G	98.2 L	98.3 G	72.8 L	96.6 N	96.5 A
0.1S	0.1S	0.1 K	0.1 M	0.1 S	0.05 I	0.05 K	0.1 A	0.1 S	0.05 V	0.1H	0.1 S
1.2 F	0.05 P	0.1 N	0.1 N		0.1 G	0.05 Q	0.05 C		0.05 D	0.05 S	0.1 T
0.4 I	0.15T	0.1 P	0.3 H		0.05 C	0.2D	0.05 I		17.8I		0.05 A
0.45V	0.15V	0.15 E	4.4 Q		0.05 R	1.6N	0.45S		0.7M		
		1.45G	1.05S		0.2A	0.15R			5.55V		

377	378	379	380	381	382	383	384	385	386	387	388
98.3 L	100 A	88.2 A	99.2 A	99.2 L	100 R	97.5G	0.857 M	100 G	94.1V	99.2 N	100 A
0.84F		0.84N	0.84T	0.84F		0.84A	0.84A		2.52I	0.84T	
		5.88S				0.84N	8.4L		3.36L		
		1.68T				0.84S	5.04T				
		1.68V									

377	378	379	380	381	382	383	384	385	386	387	388
70.1 I	99.7 A	90.6S	86 K	99.7 L	100R	98G	98M	100G	97.7 L	99.7 N	100A
16.7L	0.255T	3.06A	0.255I			2.04S	0.255I		0.255F	0.255Y	
6.58M		0.255C	1.02Q				1.53L		1.75I		
13.26V		6.12T	12.5R				0.255T		0.255P		

389	390	391	392	393	394	395	396	397	398	399
99.9V	99.9 A	99.9 Y	99.9Y	99.9 R	99.9 G	99.9 L	99.9 D	99.9 V	99.9S	98.8 V
	0.05V	0.05F		0.05C	0.05A	0.05R	0.05V	0.05M		0.1A
										0.05I
										1T

389	390	391	392	393	394	395	396	397	398	399
97.4 V	96.1 A	97.9 Y	99.2 Y	97.5 R	96.4 G	97.1 L	98.4 D	99.0 V	98.7 S	97.4 V
0.1 A	0.05 S	0.1 R	0.1 I	0.1 T	0.1 S	0.1 V	0.05 L	0.05 A	0.05 C	0.1 T
0.05 P	0.15V	0.05 C	0.05 C			0.05 A	0.05 N	0.05 M	0.05 Y	0.1 P
0.05 L		0.3 F				0.05 C		0.05 T	0.25 A	0.05 A
0.95I										1.35I

389	390	391	392	393	394	395	396	397	398	399
100 V	99.2 A	93.3 Y	100 Y	100 R	100 G	100 L	100 D	100 V	100 S	93.3 V
	0.84S	6.7F								6.7I

389	390	391	392	393	394	395	396	397	398	399
100V	95.9 A	99 Y	99.5 Y	99.7 R	99.7 G	100L	99.7 D	100V	100S	86.7V
	0.51S	1.02F	0.51 C	0.255K	0.255S		0.255N			0.51 A
	3.57E									12.7 I

400	401	402	403	404	405	406	407	408	409	410	411
99.9I	99.9P	89.9 T	98.8 S	99.9G	99.3 D,0.65N	99.9 V	99.3 V	99.8 V	99.9 V	99.8 A	99.9T
		10A	1.15 N			0.05I	0.65 I	0.05A	0.05L	0.1S	
		0.05P						0.1I		0.1T	
		0.05S									

400	401	402	403	404	405	406	407	408	409	410	411
99.8 I	97.5 P	76.1 T	91.5 S	97.9 G	91.9 D	98.8 V	98.4 V	98 V	98.2 V	99.1 A	98 T
0.1 S	2 Y	10.05 A	0.1 A	0.05 A	0.05 E	0.1 T	0.1 S	0.1 I	0.1 S	0.05 S	0.1 Q
0.05 L	0.05 L	0.05 H	0.1 L	0.05 S	0.05 G	0.15A	0.05 A	0.1 L		0.1W	
		0.05 D	0.05 C		7N		0.7I	0.05 A			
		0.05 N	0.3 D		0.15S						
		0.05 R	0.5G								

400	401	402	403	404	405	406	407	408	409	410	411
100 I	100 P	0.815 T	100 Q	100 G	100 D	99.2 V	100 V	99.2 V	100 V	100 A	100 T
		11.68A				0.84L		0.84I			
		7.56L									
		0.84P									

400	401	402	403	404	405	406	407	408	409	410	411
99.5 I	100P	98T	73.3T	100G	99.5 D	100V	100V	99.5 V	100C	99.7 A	100T
0.51L		0.765A	13.4A		0.51N			0.51 I		0.255T	
		0.51P	7.14S								
		0.51S	5.86V								

412	413	414	415	416	417	418	419	420	421	422	423	424
99.9D	99.9A	99.9L	99.9M	99.9 T	99.9G	73.6 F	99.9 T	99.9G	100D	99.9F	100D	100S
				0.05A		26.95Y	0.05A					

412	413	414	415	416	417	418	419	420	421	422	423	424
99.3 D	98.3 A	96.9 L	99.8 M	98.8 T	98.8 G	70.9 F	98.1 T	98.8 G	97.9 D	98.2 F	98.6D	98.1 S
0.05 L	0.1 T	0.05 P	0.05 N	0.05 D	0.05 R	0.05 L	0.05 A	0.05 P	0.05 A	0.05 L	0.1G	0.1 S
0.05 Q	0.05 R	0.05 S				0.05 S	0.05 H	0.05 R	0.05 R	0.05 N	0.05Y	0.05 L
0.05 R						23.2Y	0.3 S		0.3 N	0.05 S		0.05 T

412	413	414	415	416	417	418	419	420	421	422	423	424
99.2 D	100 A	100 L	100 M	99.2 T	100 G	0.798 Y	96.6T	100 G	99.2 D	100 F	100 D	100 S
				0.84A		20.16F	3.36S		0.84N			

412	413	414	415	416	417	418	419	420	421	422	423	424
99.7 D	99.5 A	100L	100M	99.5 T	100G	70.765F	98.7 T	99.5 G	100D	99.7 F	99.7 D	99.7 S
0.255G	0.51V			0.51 A		26.53Y	0.765A	0.255R			0.255E	0.255T
							0.51I					

425	426	427	428	429	430	431	432	433	434	435	436	437	438
99.8 V	99.9 I	100D	100C	100N	99.9T	100C	100V	100T	100Q	99.85T	100V	100D	99.9F
0.2 L	0.1V				0.05M					0.1A			0.05L
										0.05I			

425	426	427	428	429	430	431	432	433	434	435	436	437	438
98.2 V	98.6I	99.6 D	95 C	95.2 N	96.5 T	98.3 C	98.8 V	94.1 T	98.3 Q	98 T	98.6V	96.7 D	98.6F
0.05 R	0.1 T	0.05 G	0.05 L	0.1 A	0.1 A	0.05 L	0.1 T	0.1 N	0.05 P	0.05 N	0.1 T	0.05 A	0.1 I
0.05 S	0.05 A	0.05 R	0.05 R		0.1L	0.05 M	0.05 C	0.05 H	0.1 R	0.05 V	0.05 A	0.05 V	0.1 L
0.05Q	0.05 D	0.05 S	0.1P		0.05 Y	0.05 R	0.05 I	0.05 Q		0.1A	0.05 D	0.3 E	
	0.15V	0.05 L			1.4V	0.25S		0.05 R		0.2S	0.05 S		
								0.05 A					

425	426	427	428	429	430	431	432	433	434	435	436	437	438
100 V	99.2 I	100 D	99.2 C	100 N	98.3 V	100 A	100 V	97.5T	100 Q	0.849 I	100 V	100 D	100 F
	0.84V		0.84R		1.68T			2.52S		2.52A			

425	426	427	428	429	430	431	432	433	434	435	436	437	438
100V	99.2 I	100D	100C	99.5 N	100V	98.2 A	100V	99.7 E	100Q	100Y	99.7 V	100D	100F
	0.765V			0.51S		1.27T		0.255G			0.255A		
						0.51V							

439	440	441	442	443	444	445	446	447	448	449	450	451	452
100S	100L	99.9D	100P	99.9T	99.9F	99.9T	100I	99.8 E	99.9T	99.75 T	100T	98.3 L	100P
		0.05Y		0.05S	0.05L	0.05H		0.15D	0.05I	0.05A		0.05A	
								0.05G		0.2I		1.7 V	

439	440	441	442	443	444	445	446	447	448	449	450	451	452
98.8 S	97.1 L	98.4 D	98.2 P	98.8 T	98.8 F	98.9 T	97.2 I	97.5 E	97.6 T	95.5 T	98.4 T	94.7 V	98 P
0.05 H	0.1 V	0.1 S		0.1R	0.05 L	0.05 H	0.1V	0.1 S	0.05 D	0.1 V	0.1 V	0.1 P	0.1 A
0.05 Q	0.15F	0.05 G		0.05 Y	0.05 Q	0.25 A	0.05 H	1.15D	0.6A	0.05 I	0.05 A	0.05 I	
0.2 G	0.2M	0.05 H			0.05 R	0.5S	0.05 M			0.05 N	0.05 D	0.05 R	
0.05T							0.05 T			0.05 R	0.05 I	1.7L	
										0.35A		1.65M	

439	440	441	442	443	444	445	446	447	448	449	450	451	452
100 S	100 L	100 D	100 P	100 T	100 F	99.2 T	98.3 I	99.2 T	98.3 T	100 Q	99.2 T	99.2 V	100 P
						0.84S		0.84A	0.84I		0.84I	0.84A	
									0.84V				

439	440	441	442	443	444	445	446	447	448	449	450	451	452
100S	99 L	100D	100P	100T	99.7 F	100S	100I	100E	99.2 T	98R	99.7 T	99.5 A	100P
	1.02M				0.255L				0.51 A	0.51 C		0.51V	
									0.255I	1.53H			

453	454	455	456	457	458	459	460	461	462	463	464	465	466
100Q	99.9D	99.9A	100V	99.9S	100R	98.3 T	99.75 Q	100R	100R	99.9 G	100R	99.9T	100G
	0.05N	0.05S		0.05T		1.75 S	0.25 H			0.05D		0.05A	
										0.05S			

453	454	455	456	457	458	459	460	461	462	463	464	465	466
97.7 Q	98.2 D	97.2 A	98.4 V	97.9 S	99.3 R	93.4 S	97.8 Q	98.3 R	98 R	97.9 G	98.5 R	98.7 T	96.5 G
0.1 S	0.1 P	0.1 L	0.1 R	0.1 A	0.1 A	0.1 A	0.15A	0.1 P	0.1 Q	0.1 T	0.05 K	0.05 A	0.05 S
0.15P	0.05 G	0.15R	0.05 S	0.1 I	0.05 T	0.05 H	0.1C	0.05 L	0.05 A	0.05 E	0.05 Q	0.05 N	0.05 W
	0.1R	0.35S	0.1G	0.1 R		0.15P		0.05 T					0.1C
				0.1V		4.2T		0.1G					
				0.05 C									

453	454	455	456	457	458	459	460	461	462	463	464	465	466
100 Q	98.3 D	100 A	100 V	100 S	100 R	99.2 S	100 Q	100 R	98.3 R	100 G	100 R	100 T	100 G
	1.68E					0.84I			1.68T				

453	454	455	456	457	458	459	460	461	462	463	464	465	466
100Q	99.7 D	99.7 A	100V	99.5 S	99.5 R	99.7 S	99.2 Q	100R	99.5 R	100G	100R	99.7 T	100G
				0.51 A	0.255H		0.51R		0.51 H			0.255A	
					0.255L								

467	468	469	470	471	472	473	474	475	476	477	478	479	480
99.9R	100G	77.8 K	98.2P	100G	98.9 I	100Y	99.9R	100F	99.9V	93.7A	99.9 P	100G	99.9E
0.05M		21.1R	1.1 G		1.1T		0.05G		0.05M	6.3 T	0.05L		0.05G
			0.65R								0.05S		
			0.05S										

467	468	469	470	471	472	473	474	475	476	477	478	479	480
98.9 R	98.6G	96.1 R	78.7 R	98.5 G	0.928 I	97.8 Y	98.2 R	95.8 F	98.8 V	0.928 T	98.3 P	98.5 G	97.4 E
0.05 S	0.1 A	0.05 Q	8.55 A	0.05D	0.1 E	0.05 A	0.05 E	0.05 V	0.1 A	0.1G	0.05 A	0.05 R	0.05 R
0.05 K	0.05 S	0.05 T	0.05 D		0.05 H	0.05 H	0.05 G	0.1L	0.05 C	0.05 D	0.05 S		0.05 V
		2.8k	0.15E		0.05 M	0.05 L	0.05 M	2.25Y	0.05 I	5.4A	0.05 V		
			0.05 L		0.05 S	0.05 V	0.05 Q			0.1I			
			0.05 M		2.1T					0.3 S			

467	468	469	470	471	472	473	474	475	476	477	478	479	480
100 R	100 G	99.2 R	97.5L	99.2	0.597 I	100 Y	100 R	99.2 Y	100 V	99.2 S	92.4S	100 G	100 E
		0.84Q	0.84P	0.84V	59.66I					0.84P	3.36T		
					2.52 L						4.2P		
					26.89T								
					10.9 V								

467	468	469	470	471	472	473	474	475	476	477	478	479	480
100R	99.7 G	99.7 R	90.3L	99.7 G	94.9 T	100Y	99.5 R	99.5 Y	99.2 V	71T	98.2 P	99.7 G	100E
	0.255R	0.255K	0.255H	0.255S	0.51 A		0.255Q	0.51 F	0.765A	0.255G	0.255A	0.255D	
			9.43P		3.57I					0.255N	0.255L		
					0.255M					2.29S	1.27S		
					0.51V					26.22A			

481	482	483	484	485	486	487	488	489	490	491	492	493	494
100R	100P	100S	100G	96.1	100F	99.9D	100S	99.9S	76,5 VV	99.9L	100C	100E	99.9 C
				3.85 I		0.05V		0.05F	25.5 I	0.05F			0.1S
				0.05V									

481	482	483	484	485	486	487	488	489	490	491	492	493	494
98.9 R	97.9 P	98.7 S	98.5 G	99.6 M	98.2 F	97.5 D	97.4 S	98 0.1 S	96.2 V	97.7 L	97.9 C	99.4 E	98.8 C
0.1 A	0.1 S	0.1 T	0.05 A	0.2L	0.05 L		0.05 F	0.05 L	0.1 A	0.05 P	0.05 M	0.1 D	0.1 A
0.05 T	0.05 A	0.15L		0.05 M	0.05 V		0.35T	0.25 A	0.05 G	0.15I			0.05 L
	0.15 T			0.05 H				0.4V	0.05 T				0.05 S
				0.05 S					2.65I				0.05 V

481	482	483	484	485	486	487	488	489	490	491	492	493	494
99.2 R	96.6P	100 S	99.2	98.3 M	99.2 F	99.2 D	96.6S	93.3 V	97.5V	99.2 L	99.2 C	97.5E	98.3 C
0.84S	1.68S		0.84A	0.84V	0.84L	0.84H	0.84A	1.68A	0.84G	0.84I	0.84S	0.84Y	1.68F
	1.68A			0.84R			0.84R	1.68E	0.84I				
							1.68G	0.84G	0.84M				
								0.84I					

481	482	483	484	485	486	487	488	489	490	491	492	493	494
98.7 R	99.2 P	100S	100G	99.5 M	100F	100D	100S	92.6 V	99.5 V	99.7 L	100C	99.7 E	99.5 C
1.27K	0.255L			0.255L				7.14A	0.51 I	0.255P		0.255G	0.51Y
				0.255V				0.255S					

495	496	497	498	499	500	501	502	503	504	505	506	507	508
100Y	99.9D	100A	99.9 G	100C	100A	100W	100Y	100E	100L	99.9 T	99.8 P,	97.6 A	100E
	0.05N		0.01S							0.05A	0.05A	22.5S	
										.05M	0.15 S	0.1T	

495	496	497	498	499	500	501	502	503	504	505	506	507	508
99.7 Y	99.8 D	98.4 A	98.7 G	98.3 C	99.2 A	99.8 W	96.5 Y	99.0 E	98.5 L	97.7 T	98.5 P	97.6 A	99.4 E
0.05 L	0.1E	0.1 N		0.1 P	0.1 R	0.1 A	0.1 H	0.1 S	0.1 I	0.05 A	0.1 R	0.05 R	0.1 C
0.05 R		0.1 T		0.05 L	0.05 C	0.05 L	0.05 V	0.05 D	0.05 A	0.05 P	0.05 A	0.65S	0.05 K
0.05 Q		0.05 V		0.05 R	0.05 T	0.05 R	0.2C	0.05 G		0.05 H	0.05 P		0.05 R
		0.05 R			0.2S		0.15F			0.05 L			
		0.4S								0.05 M			

495	496	497	498	499	500	501	502	503	504	505	506	507	508
99.2 Y	99.2 D	94.1A	99.2	100 A	99.2 A	100 W	100 Y	96.6E	100 L	96.6T	100 P	95.8 A	100 E
0.84R	0.84Y	4.2S	0.84R		0.84S			3.36D				4.2S	
		1.68T											

495	496	497	498	499	500	501	502	503	504	505	506	507	508
100Y	100D	98.2 A	92.9 G	99.5 C	97.7 S	100W	100Y	99.2 D	100L	100Q	99.7 P	98.7 A	100E
		1.53T	0.51T	0.255Y	2.29A			0.765E			0.255L	0.255G	
												0.255P	
												0.765S	

509	510	511	512	513	514	515	516	517	518	519	520	521	522
99.9T	98.3 T	100V	100R	100L	100R	99.7A	100Y	93.6M	95.3 N	99.9T	99.9P	100G	100L
0.05A	1.75S					0.25S		3.85F	4.6D	0.05A	0.05L		
						0.05T		2.5 L	0.05H				
								0.05I					

509	510	511	512	513	514	515	516	517	518	519	520	521	522
99. T	89.35S	95.9 V	97.3 R	95.7 L	99.4 R	97.5 A	99.7 Y	97.0 L	94.7 N	99 T	98.5 P	99.2 G	97.4 L
0.1 V	8.03T	0.1 A	0.05 M	0.05V	0.05 A	0.05 G	0.05V	0.1 I	0.1G	0.1 A	0.05 T	0.1 S	0.1 R
0.05 A	0.04H	0.05 I		0.1 T			0.05 L	0.05 L	0.05 S	0.05 Y		0.05 R	0.05 F
0.05 D	0.04P	0.05 S						0.05 P	0.05 K				0.05 V
0.05 I													

509	510	511	512	513	514	515	516	517	518	519	520	521	522
100 T	100 T	96.6V	99.2 R	100 L	99.2 R	100 A	100 Y	99.2 F	99.2 N	99.2 T	100 P	100 G	100 L
		3.36G	0.84T		0.84P			0.84L	0.84T	0.84A			

509	510	511	512	513	514	515	516	517	518	519	520	521	522
99 T	99.2 T	99.5 V	100R	100L	99.2 R	99.5 A	99.7 Y	100L	99 S	99.7 T	100P	100G	99.5 L
0.255V	0.51 A	0.51 A			0.51 G		0.255C		0.51 N				
0.765I	0.255I				0.255S				0.51T				

523	524	525	526	527	528	529	530	531	532	533	534	535	536
99.9P	100V	99.9 C	99.6 Q	100D	100H	100L	99.9E	100F	100W	100E	98.2G	100V	99.9 F
0.05L		0.1S	0.4 H				0.05G				1.8 S		0.1L

523	524	525	526	527	528	529	530	531	532	533	534	535	536
99.8 P	96.4 V	98.4 C	99.0 Q	98.8 D	97.0 H	98.5 L	99.3 E	98.4 F	99.8 W	99.6 E	84.77S	98.9 V	96.8 F
0.05 A	0.1F	0.1 R	0.05M	0.1 R	0.1 L	0.1G	0.1 C	0.1 G		0.05 E	12.21G	0.05 G	0.05 H
0.05 T	0.05 L	0.1W	0.05 P	0.05 G	0.05 H	0.05 P	0.05 G	0.05 L		0.1 T	0.22A	0.05 A	0.05 S
	0.05 P	0.05 L	0.05 S		0.05 Y			0.05 V		0.05 G	0.04D	0.05 L	0.05Y
	0.05 R	0.05 V			0.05 P							0.05 R	
	0.05 I	0.05 S											

523	524	525	526	527	528	529	530	531	532	533	534	535	536
100 P	100 V	99.2 C	100 Q	100 D	100 H	100 L	100 E	100 F	100 W	96.6E	97.5A	100 V	100 F
		0.84H									2.52G		

523	524	525	526	527	528	529	530	531	532	533	534	535	536
100P	100V	99 C	98.7 Q	99.7 D	100H	99.2 L	98.5 D	99.5 F	99.7 W	100E	96.2 S	98.2 V	99.5 F
		0.255W	0.255H				1.53E	0.51L	0.255R		3.31G	1.53I	0.51Y
		0.255Y	1.02R								0.51N		

537	538	539	540	541	542	543	544	545	546	547	548	549	550
99.8 T	100G	100L	100T	96.4 H	99.9 I	100D	100A	100H	99.9F	100L	100S	100Q	99.9 T
0.15A				3.6 R	0.05M				0.05Y				0.1A
0.05M					0.05V								

537	538	539	540	541	542	543	544	545	546	547	548	549	550
97.6 T	99.5 G	99.3 L	98 T	96.5 H	98.5 I	96.5 D	97.9 A	96.5 H	97.9 F	97.3 L	99.0 S	98.4 Q	96.4 T
0.1 Q	0.05 R	0.05 C	0.05V	0.05 F	0.1 A	0.05 N	0.05 P	0.1 I	0.05 C	0.1 S	0.05 F	0.1G	0.1 I
0.1 S		0.05 P	0.05 H	0.05 P	0.05 H	0.1 R	0.05 R	0.05 F		0.05 Q	0.05 V	0.05 P	0.05 D
0.05 A		0.05 Y		0.05 K		0.05 E	0.05 S	0.05 P		0.05 M			0.05 N
0.05 H				0.05 R		0.05 W							0.05 V
0.05 K				0.05 S									

537	538	539	540	541	542	543	544	545	546	547	548	549	550
100 T	99.2	100 L	94.1T	100 H	100 I	99.2 D	100 A	100 H	100 F	99.2 L	100 S	100 Q	99.2 T
	0.84S		1.68R			0.84V				0.84P			0.84N
			0.84S										

537	538	539	540	541	542	543	544	545	546	547	548	549	550
99.5 T	99.5 G	99.2 L	94.4 T	100H	99.7 I	100D	99.7 A	99.5 H	98.5 F	99.7 L	99.7 S	100Q	98.2 T
0.255A	0.51R	0.255P	0.51S		0.255V		0.255T	0.255Q	1.02L		0.255P		0.765A
			5.1P										

551	552	553	554	555	556	557	558	559	560	561	562	563
99.9K	99.85Q	82.2 S	99.9G	98.3E	99.9N	76.5 F	99.9P	100Y	100L	100V	100A	100Y
0.05T	0.15 H	1.75A		1.75 D	0.05T	24.45 L	0.05L					
		16.1 G										

551	552	553	554	555	556	557	558	559	560	561	562	563
96 K	99.3 Q	98.4 A	98.4 G	83.79D	98.5 N	91.9 F	98.1 P	98.9 Y	98.2 L	77.83V	98.2 A	98.8 Y
0.1 A	0.1 S	0.05 L	0.1 Q	14.61E	0.05 H	0.05 L	0.1 T	0.05 F	0.05 P	0.98A	0.05 V	0.1 S
0.05 R	0.05 T		0.05 R		0.05 N		0.05 S	0.05 L		0.04G	0.05 G	0.05 C
					0.05 Q					0.84I		0.05 V
										0.22L		
										0.22T		

551	552	553	554	555	556	557	558	559	560	561	562	563
99.2 K	99.2 Q	92.4G	100 G	80.7 D	100 N	99.2 F	100 A	100 Y	100 L	85.7 T	100 A	100 Y
0.84N	0.84F	5.88A		19.32E		0.84L				0.84M		
										11.76V		

551	552	553	554	555	556	557	558	559	560	561	562	563
99.7 K	100Q	99.7 Q	100G	99.2 L	99.7 N	99.5 F	86.7S	98.2 Y	99.7 L	71.7 T	99.7 A	100Y
0.255E		0.255A		0.51I	0.255S	0.51S	3.57A	1.53F		21.4A		
							9.18 P			6.88V		

564	565	566	567	568	569	570	571	572	573	574	575	576	577
100Q	99.9A	100T	100V	100C	99.9A	100R	100A	99.9 Q	100A	99.85P	100P	100P	100S
	0.05T				0.05V			0.01R		0.05L			
										0.1S			

564	565	566	567	568	569	570	571	572	573	574	575	576	577
98.3 Q	96.9 A	97.6 T	99.3 V	98.7 C	99.0 A	96.7 R	96.1 A	94.7 Q	96.2 A	96.5 P	98 P	98.9 P	96.9 S
0.1 C	0.05 G	0.1 R	0.1 A	0.05 N	0.1 T	0.05 W	0.1 A	0.05 M	0.1 T	0.05 F	0.1L	0.1T	0.1W
0.05 P	0.05 L	0.05 A	0.1 L	0.05 S	0.05V	0.05 G	0.05 E	0.05 A	0.05 C		0.05 T	0.05 S	0.05 I
0.05 R	0.05 P	0.05 H	0.05 E	0.05 V	0.05 A		0.05 G	0.05 C	0.05 S				
			0.05 G					0.05 G					
								0.05 H					

564	565	566	567	568	569	570	571	572	573	574	575	576	577
99.2 Q	100 A	100 T	100 V	97.5C	100 A	98.3 R	100 A	95.8 K	99.2 A	99.2 P	100 P	100 P	99.2 S
0.84T				0.84W		0.84K		1.68R	0.84S	0.84L			0.84P
						0.84T		0.84T					

564	565	566	567	568	569	570	571	572	573	574	575	576	577
100Q	100A	100T	99.5 V	100C	99.7 A	99.5 R	100A	99.7 Q	99.2 A	0.901 P	100P	99.5 P	91.1 S
			0.255A		0.255S	0.255C		0.255L	0.255V	8.16L		0.255Q	8.41C
			0.255M			0.255H			0.51T	1.78S		0.255S	0.255T
													0.255V

578	579	580	581	582	583	584	585	586	587	588	589	590	591
100W	100D	100Q	99.9 M	99.9W	99.9K	100C	100L	70.65I	100R	99.9L	99.9 K	100P	100T
			0.05T	0.05R	0.05R			30.15 T		0.05I	0.05R		
			0.05V					0.05A					
								0.05L					
								0.1N					

578	579	580	581	582	583	584	585	586	587	588	589	590	591
99.9 W	968 D	96.5 Q	99.0 M	99.8 W	97.9 K	98.1 C	98.9 L	83.8 I	99.7 R	96.98L	97.3 K	98.2 P	94.7 T
0.05 V	0.05 G	0.05 E	0.1 I	0.1 L	0.1Q	0.1 C	0.05 A	0.1 P	0.1 G	0.11A	0.05 E	0.05 A	0.05 H
	0.05 H	0.05 F	0.05 A	0.05 P	0.05 E	0.05 F	0.05 S	0.05 H	0.05 T	0.04P	0.05 G	0.05 L	0.05 N
		0.05 I	0.05 K	0.05 R		0.05 L	0.05 V	0.05 N	0.05 W	0.11Q	0.05 N		0.05 S
		0.05 P	0.05 M	0.05 V		0.05 S							0.05 V
		0.05 V	0.05 N										0.05 Y

578	579	580	581	582	583	584	585	586	587	588	589	590	591
100 W	99.2 D	91.6 V	98.3 M	100 W	100 K	99.2 C	98.3 L	89.1 T	100 R	100 L	99.2 K	100 P	99.2 T
	0.84E	2.52A	1.68T			0.84R	1.68R	2.52 I			0.84R		0.84I
		0.84I						3.36N					
		0.84L						0.84S					
		1.68M						4.2A					

578	579	580	581	582	583	584	585	586	587	588	589	590	591
99.7 W	100D	99.2 E	0.638 T	100W	99.2 K	99.5 C	99.7 L	80.6 V	100R	99.7 L	99.7 K	100P	99.7 T
0.255G		0.255G	36.2M		0.765R	0.255R	0.255P	0.765A		0.255H			0.255A
		0.255K				0.255V		7.9I					
								3.31L					
								1.275M					
								6.12T					

592	593	594	595	596	597	598	599	600	601	602	603	604	605
100L	99.85H	100G	99.9 P	100T	100P	100L	100L	100Y	100R	100L	100G	0.997A	99.85V
	0.15R		0.1S									0.05G	0.05A
												0.15S	0.1F
												0.05T	
												0.1V	

592	593	594	595	596	597	598	599	600	601	602	603	604	605
98.4 L	97.3 H	99.4 G	95.86P	96.8 T	99.1 P	98.3 L	98.69L	97.53Y	99.7 R	96.84L	99.2 G	98.3 A	96.5 V
0.05 A	0.05 N	0.1 R	0.58A	0.1 G	0.1 S	0.05 V	0.04P	0.04F	0.1 C	0.25I	0.1 R	0.1 T	0.05 E
0.05 M	0.05 A	0.05 H	0.33Q	0.05 A	0.05 T		0.04S	0.04V	0.05 K	0.07M	0.05 E		0.05 K
	0.05 L		1.67S	0.05 N			0.04A			0.04S			0.05 L
				0.05 P									0.05 V

592	593	594	595	596	597	598	599	600	601	602	603	604	605
100 L	0.908 T	100 G	100 P	100 T	100 P	100 L	100 L	100 Y	99.2 R	100 L	100 G	95.0 A	98.3 V
	1.68A								0.84P			1.68P	1.68I
	0.84S											0.84S	
	5.04S											0.84T	

592	593	594	595	596	597	598	599	600	601	602	603	604	605
94.9 L	93.1 H	100G	100P	100T	99.5 P	99.7 L	100L	100Y	100R	99.7 L	100G	97.7 P	70.7 V
0.255V	6.6Y				0.51R					0.255M		0.255A	29.33I
4.84M												1.785S	

606	607	608	609	610	611	612	613	614	615	616	617	618
99.9 Q	99.85N	99.6 E	79.5 V	99.8 T	100L	99.9 T	100H	99.9P	750 V	99.5 T	99.9K	98.9 Y
0.1 H	0.1 D	0.4D	20.5I	0.2 I		0.05A		0.05S	25.I	0.45 S	0.05R	1.1 F
	0.05S					0.05P			0.1 A	0.05P		

606	607	608	609	610	611	612	613	614	615	616	617	618
97.9 Q	96 N	97.3 E	77.68V	79.21T	97.35L	97.5 T	98.22T	97.96P	76.92I	97.67T	98.62K	90.77Y
0.1 P	0.05 A	0.05 K	0.04G	0.95A	0.04C	0.05 L	0.04P	0.04L	0.15K	0.8C	0.04H	7.31F
0.05 R	0.05 N	0.05 G	8.72I	0.04C	0.76F	0.05 P	0.55S	0.04N	1.67M	0.04N	0.04N	0.07H
0.05 T	0.05 Q	0.05 H	1.05L	0.04H	0.07I			0.04Q	0.04Q		0.04Q	0.04I
			0.07P	11.96I	0.04Y				0.04T		0.04T	1.09L
			10.14T	1.31N	0.18P				18.39V			

606	607	608	609	610	611	612	613	614	615	616	617	618
94.1T	99.2 N	97.5E	94.1V	100 T	99.2 L	100 T	99.2 H	100 P	98.3 V	100 T	99.2 K	100 Y
1.68A	0.84K	2.52D	5.88I		0.84M		0.84P		1.68G		0.84R	
4.2S												

606	607	608	609	610	611	612	613	614	615	616	617	618
99.5 Q	93.4 N	99 E	73.8I	100C	95.2 L	99.5 T	100H	99.5 P	70.1 V	100T	100K	100Y
0.255H	6.6H	1.02D	0.255F		0.765F	0.51 S			30 I			
			0.255L		0.255M							
			14.6T		2.04S							
			10.45V		0.51T							
					02V							

619	620	621	622	623	624	625
99.8 I	99.9M	98.2 T	100C	100M	100S	99.9A
0.2V	0.05T	0.05I				
		1.8 A				

619	620	621	622	623	624	625
98.4 I	97.56M	92.95A	99.2C	99.93M	0.95A	97.5 A
0.05 N	0.69A	0.04G	0.04A	0.04G	0.04P	0.1 C
0.05 S	0.04G	0.04H	0.04M	0.04H	98.22S	0.05 V
0.05 G	0.04H	6.65T	0.04R		0.04V	0.2P
1.05V	0.07I		0.22S		0.04W	
	0.18S		0.04V			

619	620	621	622	623	624	625
99.2 I	99.2 A	100 T	100 C	100 M	100 Q	100 A
0.84V	0.84G					

619	620	621	622	623	624	625
89.3I	87.0M	97.7 A	98.5 C	100M	98.5 S	100A
0.765L	0.255A	2.29T	.51R		1.27A	
0.255N	0.255K		0.255Y			
9.69V	41.53T		0.765S			
	0.51V					

Supplementary Figure 1: Ramachandran plots for each NS3 structure: Ramachandran plot analysis was performed for all genotype-specific structures. Plots before and after minimization are given.

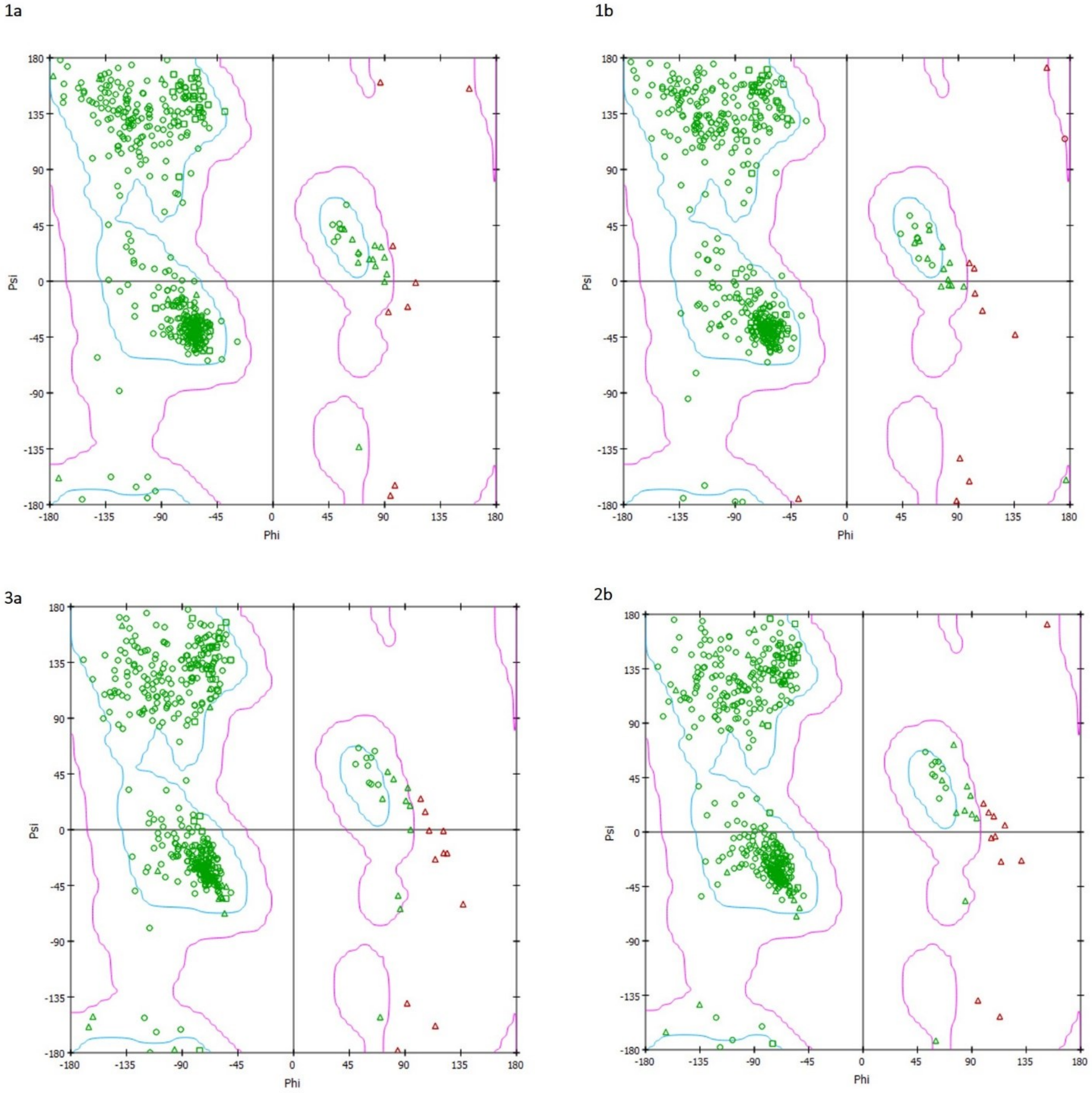

Supplementary Table 2: NS3 HCV of genotype 1a, 1b, 2b and 3a amino acids interaction with Fluoroquinolones: Molegro Virtual Docker software was employed to dock the panel of 8 fluoroquinolones (Sparfloxacin, balofloxacin, Enrofloxacin, Ofloxacin, Pefloxacin, Lomefloxacin, Levofloxacin, and Ciprofloxacin) on each of the four NS3 helicases from different HCV genotypes. The table shows amino acid residues (bold) in the fluoroquinolone binding region of NS3 helicase from different genotypes that formed interactions with the fluoroquinolones. Absence of a reside in genotype(s) is indicated by '-'. Below each amino acid nature of interaction is described, where B = backbone interaction; C = sidechain interaction; H = hydrogen bond; H-weak = weak hydrogen bond; Pi = π; F = hydrophobic; X = halogen bond; bump = Stearic (VW) clashes; and π-H = Pi donor H-bond.

Drugs	1a	1b	2b	3a
**BALOFLOXACIN**	**-**	**-**	**-**	**ASN229**
				[H:(2S)]
	**-**	**-**	**PRO230**	**PRO230**
			[H:(1B); H-weak:(3B); F:(6S)]	[H:(1B); π:(2S); F:(5S); X:(1B)]
	**-**	**-**	**GLU291**	**GLU291**
	-	-	[H:(2S); H-weak:(3S)]	[H:(1S); H-weak:(1B); X:(1S); π:(1S); F:(2S)]
	**HIS293**	**HIS293**	**HIS293**	**HIS293**
	[H:(1B) (1S); π:(2S)]	[H-weak:(1S); π:(3S)]	[H-weak:(1S); π:(3S)]	[π:(2S); H:(1B); H-weak:(1B)],
	**THR295**	**THR295**	**VAL295**	**GLN295**
	[H:(1B) (3S)]	[H:(2B); H-weak:(1S); F:(1B); X:(1B)]	[H:(3B); H-weak:(2B); π:(7S); F:(4S); π-H:(1S)]	[H:(2B); H-weak:(1S); F:(1B); X:(1B)]
	**-**	**ASP296**	**ASP296**	**ASP296**
		[H:(2B); H-weak:(3S); π:(1S); X:(1S)]	[H: (1B) (1S); H-weak:(4S); π:(7S)]	[H:(2B); H-weak:(3S); π:(1S); X:(1S)]
	**-**	**SER297**	**-**	**-**
		[H:(2B)]		
	**-**	**THR298**	**-**	**-**
		[H:(3S); π:(1S)]		
	**ARG393**	**-**	**-**	**ARG393**
	[H:(1B)(1S)]			[π-H:(1S); π:(1S); F:(2S)]
	**THR411**	**-**	**-**	**-**
	[H:(2S)]			
	**-**	**-**	**ASP412**	**-**
			[H:(1S); H-weak:(1B); π:(1S)]	
	**-**	**-**	**MET415**	**-**
			[H:(1B); H-weak:(5B); F:(4S)]	

	**VAL432**	**-**	**VAL432**	**-**
	[H:(2B); F:(2S); π:(1B)]		[H:(1B); H-weak:(1B)]	
	**-**	**-**	**-**	**GLU433**
				[π:(1B); H:(2S); H-weak:(4S)],
	**-**	**GLN434**	**GLN434**	**GLN434**
		[H:(2B)]	[H:(2B)]	[H:(3B)]
	**LEU451**	**-**	**-**	**-**
	[H:(6B); F:(1S)]			
	**PRO452**	**-**	**-**	**-**
	[H:(3B)]			
	**-**	**ASP454**	**-**	**-**
		[H:(2S); H-weak:(2S)],		
	**SER457**	**-**	**-**	**-**
	[H:(3S)]			
	**-**	**-**	**-**	**GLN460**
				[H:(1S)]
	**-**	**-**	**-**	**ARG464**
				[H:(1S)]
	**-**	**-**	**-**	**ARG467**
				[H:(3S)]
	**ARG481**	**ARG481**	**ARG481**	**-**
	[H:(6S); X:(1S)]	[H:(2S)]	[H:(2S)]	
	**GLY484**	**GLY484**	**-**	**-**
	[H:(1B)]	[H:(2B)]		
	**MET485**	**MET485**	**-**	**-**
	[H:(1B); X:(1B)]	[H:(1B); X:(1B)]		
	**-**	**-**	**-**	**ASP487**
				[H:(3B)]
	**-**	**GLU493**	**-**	**-**
		[H:(1B)]		
	**-**	**GLY554**	**-**	**-**
		[H:(1B)]		
**CIPROFLOXACIN**	**-**	**-**	**-**	**THR206**
				[H:(1S)]
	**-**	**-**	**ASN229**	**-**
			[H:(3S)]	
	**-**	**GLU291**	**-**	**-**

		[H:(1S); H-weak:(1S); π:(1S); F:(1S); X:(1S)]		
	**HIS293**	**HIS293**	**-**	**-**
	[H:(4B) (1S); π:(1S)]	[H:(1B)(1S); H-weak:(1B); π:(1S)]		
	**THR295**	**-**	**VAL295**	**GLN295**
	[H:(1B) (1S)]		[H:(3B); π:(5S); F:(2S); X:(1B)]	[H:(3B) (2S); H-weak:(1B) (3S)]
	**ASP296**	**ASP296**	**-**	**ASP296**
	[H:(1S); π:(1S)]	[H: (1S); H-weak:(1S); π:(1S)]		[H:(2S); H-weak:(1S); F:(4S); π:(2S)]
	**-**	**SER297**	**-**	**-**
		[H:(2B)]		
	**-**	**-**	**-**	**ARG393**
				[H:(1B) (1S); F:(2S)]
	**THR411**	**-**	**-**	**THR411**
	Unclassified interaction			[H:(1S)]
	**-**	**-**	**ASP412**	**-**
			[H:(1S); H-weak:(3B); π:(1S)]	
	**ALA413**	**-**	**-**	**-**
	Unclassified interaction			
	**-**	**-**	**MET415**	**MET415**
			[H:(1B); H-weak:(3B); π:(6S); F:(2S); X:(1B)]	[H:(1B); H-weak:(8B); π:(4S); F:(3S)]
	**VAL432**	**VAL432**	**-**	**-**
	[H:(3B); π:(2B)]	[H:(1B); F:(1S)]		
	**-**	**THR433**	**THR433**	**-**
		[H:(2S); H-weak:(2S)]	[H:(1S)]	
	**-**	**GLN434**	**GLN434**	**GLN434**
		[H:(3B) (1S)]	[H:(4B); H-weak:(1B)]	[H:(3B)]
	**LEU451**	**-**	**-**	**-**
	[H:(6B)]			
	**PRO452**	**-**	**PRO452**	**-**
	[H:(2B)]		Unclassified interaction	
	**ASP454**	**-**	**-**	**-**
	[H:(5S); π:(10S)]			
	**VAL456**	**-**	**-**	**-**
	[F:(10S); π:(2S)]			
	**SER457**	**-**	**-**	**-**

	[H:(1S)]			
	**-**	**GLN460**	**GLN460**	**GLN460**
		[H:(1S); H-weak:(1S)]	[H:(1S)]	[H:(2S)]
	**ARG461**	**-**	**-**	**-**
	[H:(2S)], one bump was also observed in pose 02			
	**-**	**-**	**-**	**ARG467**
				one H-bond with side chain is possible
	**ARG481**	**-**	**-**	**-**
	[H:(8S)]			
	**GLY484**	**GLY484**	**-**	**-**
	[H:(1B)]	[H:(1B)]		
	**MET485**	**MET485**	**-**	**-**
	[H:(3B)]	[H:(3B); H-weak:(1B)]		
	**-**	**-**	**ASP487**	**ASP487**
			[H:(1S)]	[H:(2B)]
	**-**	**-**	**GLU493**	**-**
			[H:(1B); H-weak:(1S)]	
	**-**	**TRP501**	**-**	**-**
		[H:(1S); H-weak:(1S); π:(8S)]		
	**-**	**ASP555**	**-**	**-**
		[H:(1B); H-weak:(1B)],		
	**-**	**ASN556**	**-**	**-**
		[H:(1B); H-weak:(1S) with two bumps],		
	**-**	**-**	**PHE557**	**-**
			[H:(1B)]	
**ENROFLOXACIN**	**-**	**-**	**-**	**ASN229**
				[H:(3S)]
	**-**	**-**	**-**	**SER231**
				[H-weak:(1S)]
	**-**	**-**	**GLU291**	**GLU291**
			[H:(1S); H-weak:(6S)]	[H:(1S); H-weak:(3S); F:(2S)]
	**-**	**HIS293**	**-**	**-**
		[H-weak:(1S); π:(3S)]		
	**THR295**	**THR295**	**VAL295**	**-**

	[H:(1S)], H-weak(5B) (2S)]	[H:(2B); H-weak:(3S)]	[H:(3B); H-weak:(1B); π:(3S); F:(7S); π-H:(1B)]	
	**-**	**SER297**	**-**	**-**
		[H:(2B)]		
	**-**	**THR298**	**-**	**-**
		[H:(3S)]		
	**THR411**	**-**	**-**	**-**
	Unclassified interaction			
	**-**	**-**	**MET415**	**MET415**
			[H:(1B); H-weak:(7B); π:(6S); F:(6S)],	[H:(3B); H-weak:(2B); F:(8S); π:(5S)]
	**-**	**-**	**VAL432**	**-**
			[H:(1B)],	
	**-**	**-**	**-**	**GLU433**
				[H:(1S); F:(3B)]
	**-**	**-**	**-**	**GLN434**
				[H:(2B)]
	**LEU451**	**-**	**-**	**-**
	[H:(2B)]			
	**PRO452**	**-**	**-**	**-**
	[H:(1B)]			
	**ASP454**	**ASP454**	**ASP454**	**ASP454**
	[H:(1B) (1S); π:(5S)]	[H:(1B) (1S); F:(1S)]	[H:(2B) (1S); H-weak:(3S)]	[H:(1B) (1S); F:(1S)]
	**SER457**	**-**	**-**	**SER457**
	[H:(1B) (3S)]			[H:(1S); H-weak:(1B); F:(1S)]
	**GLN460**	**GLN460**	**GLN460**	**GLN460**
	[H:(2S)], H-weak(4S)]	Unclassified interaction	Unclassified interaction	Bump
	**ARG464**	**-**	**-**	**-**
	[H:(1S)]			
	**ARG481**	**ARG481**	**ARG481**	**ARG481**
	[H:(6S)]	[H:(4S)]	[H:(4S)]	[H:(1S)]
	**-**	**GLY484**	**-**	**-**
		[H:(2B)]		
	**MET485**	**MET485**	**-**	**-**
	[H:(3B)]	[H:(3B); H-weak:(1B)]		
	**ASP487**	**-**	**-**	**ASP487**
	Unclassified interaction			[H:(3B)]

	-	**GLU493**	-	-
		[H:(1B)]		
	-	**TRP501**	-	-
		[H:(2S); H-weak:(1S); π:(2S)]		
	-	**TYR502**	-	-
		Unclassified interaction		
	-	**ASN556**	-	-
		[H:(1S)]		
**LOMIFLOXACIN**	-	-	-	**PRO230**
				[H:(1B); X:(1B)]
	-	-	**GLU291**	-
			[H:(2S); H-weak:(2S); X:(2S)]	
	**HIS293**	**HIS293**	-	**HIS293**
	[H:(2B) (1S); π:(2S)]	[H:(2B) (2S); H-weak(1S); π:(1S)]		[π:(2S); H-weak:(1B) (1S)],
	**THR295**	-	**VAL295**	**GLN295**
	[H:(1B); H-weak(4S); π:(1S)]		[H:(2B); H-weak:(3B); π:(3S); F:(5S)]	[H:(2B); H-weak:(2S); X:(1B) (3S); π:(1S)]
	-	-	**ASP296**	**ASP296**
			[H:(1S); H-weak:(1S); π:(4S)]	[H:(1S); H-weak:(1S); F:(3S); π:(2S)],
	-	-	-	**ALA297**
				[H:(1B); π:(1S)],
	-	**HIS369**	-	-
		[H:(1B); H-weak(1B);]		
	**THR411**	-	-	-
	[H:(2S)]			
	-	**ASP412**	**ASP412**	-
		[H:(1B); H-weak:(3S)]	[H:(1S); H-weak:(1S)]	
	-	**MET415**	**MET415**	-
		[H:(3B); H-weak:(5B); X:(3B); π:(2S); F:(2S)]	[H:(2B); H-weak:(3B); X:(2B); π:(3S); F:(2S)]	
	-	-	-	**THR416**
				Unclassified interaction
	**VAL432**	-	-	-
	[H:(1B); π:(3B) (1S)]			
	**THR433**	-	**THR433**	-
	[H:(1S)]		Unclassified interaction	

	-	**GLN434**	**GLN434**	-
		[H:(3B) (3S); H-weak:(1B) (5S); π:(1B)]	[H:(2B); X:(1B)]	
	**LEU451**	-	-	-
	[H:(7B)]			
	-	**ASP454**	**ASP454**	**ASP454**
		[π:(4S)]	[H:(1B); H-weak:(1S)]	[H:(1S); H-weak:(3S); X:(1S)],
	**SER457**	-	-	-
	[H:(2S)]			
	-	-	-	**GLN460**
				[H:(1S); X:(1S)]
	**ARG461**	-	-	-
	[H:(3S); π:(2S)]			
	-	**PRO482**	-	-
		[H:(1B); H-weak(1B)]		
	**SER483**	-	-	-
	Unclassified interaction			
	**GLY484**	-	-	-
	[H:(1B)]			
	**MET485**	**MET485**	-	-
	[H:(1B)]	[H:(4B); H-weak(2B)]		
	**ARG481**	**ARG481**	**ARG481**	-
	[H:(2S); π:(1S)]	[H:(3S); π:(1S)]	H:(2S)]	
	-	**ASP487**	**ASP487**	**ASP487**
		[H:(1B); H-weak:(1B)]	[H:(1B)]	Unclassified interaction
	-	-	**GLU493**	-
			[H:(1S); H-weak:(3S)]	
	-	-	-	**ASP496**
				[H:(1S)]
	-	-	-	**TRP501**
				[H:(1S)]
	-	-	-	**SER558**
				Unclassified interaction
	-	-	-	**ASN229**
**LEVOFLOXACIN**				[H:(2S)]
	-	-	-	**SER231**
				[H:(1B); H-weak:(1S)]

	-	-	**GLU291**	**GLU291**
			[H:(1S); H-weak(2S); X:(1S)]	[H:(1S); H-weak:(2S)]
	**HIS293**	**HIS293**	-	-
	[H:(1B)(1S); π:(2S)]	[H-weak:(1S); π:(3S)]		
	**THR295**	**THR295**	**VAL295**	**GLN295**
	[H:(2B) (2S; one bump was also observed in pose 03]	[H:(2B); H-weak:(3S)]	[H:(4B); H-weak:(1B); π:(3S); F:(8S)]	[H:(2B); H-weak:(3S)]
	-	-	-	**ASP296**
				[H:(1S); H-weak:(4S); π:(1S); F:(1S)]
	-	-	-	**ALA297**
				[H:(1B); π:(1S)]
	**HIS369**	-	-	-
	Unclassified interaction			
	**ASP412**	-	-	**ASP412**
	[H:(1S), H-weak(4S)]			[H:(1S); H-weak:(3B)(1S); π:(1S); F:(S)]
	-	-	**MET415**	-
			[H:(3B); H-weak:(3B); π:(3S); F:(7S)]	
	**VAL432**	-	-	-
	[H:(1B)]			
	-	-	**THR433**	**GLU433**
			Unclassified interaction	[H:(1S); H-weak:(3S)]
	-	**GLN434**	**GLN434**	-
		[H:(2B)]	[H:(2B)]	
	**LEU451**	-	-	-
	[H:(3B) one with bump; H-weak(3B); F:(1S)]			
	**ASP454**	-	**ASP454**	**ASP454**
	[H:(1B), H-weak(4S); π:(10S)]		[H:(2B) (1S); H-weak(3S); X:(1S)]	[H:(2S); H-weak:(5S); π:(1S)]
	-	**ASP555**	-	-
		[H:(1B); H-weak:(1B)]		
	**SER457**	-	-	**SER457**
	[H:(1B) (1S); H-weak(1B) (1S); π:(1S)]			[H:(2S)]
	**GLN460**	**GLN460**	-	-
	[H:(4S), H-weak(1S)]	[H:(1S)]		

	**ARG461**	**-**	**-**	**-**
	[H:(1S); H-weak(1S)]			
	**ARG481**	**ARG481**	**ARG481**	**ARG481**
	[H:(5S)]	[H:(2S)]	[H:(2S)]	[H:(1S)]
	**GLY484**	**GLY484**	**-**	**-**
	[H:(1B)]	[H:(1B)]		
	**MET485**	**MET485**	**-**	**-**
	[H:(1B), H-weak(6B)]	[H:(1B), H-weak(6B)]		
	**-**	**-**	**-**	**ASP487**
				1 H-bond with backbone accompanied with a bump
	**-**	**-**	**-**	**TRP501**
				Unclassified interaction
	**-**	**-**	**-**	**ASN556**
				[H:(1S); H-weak:(1S); F:(1S)]
	**-**	**PHE557**	**-**	**PHE557**
		[H:(1B)]		[H:(1B)]
	**-**	**-**	**-**	**SER558**
				[H:(1B)]
**OFLOXACIN**	**-**	**-**	**ASN229**	**ASN229**
			[H-weak:(1S)]	[H:(1S)]
	**-**	**HIS293**	**-**	**HIS293**
		[H:(1B) (3S); H-weak:(8B); π:(1S)]		[H-weak:(1S); H:(1B) (1S); π:(2S); F:(1S)]
	**SER294**	**-**	**-**	**-**
	[H-weak(1B)]			
	**THR295**	**THR295**	**VAL295**	**GLN295**
	[H:(3B) (1S); H-weak(1S)]	[H:(1B) (1S); H-weak:(2B) (3S)]	[H:(2B); π:(3S); F:(7S)]	[H:(5B)(1S)]
	**-**	**ASP296**	**-**	**-**
		[H:(1S); H weak:(1S); π:(3S)]		
	**-**	**-**	**-**	**ALA297**
				[H:(1B)]
	**THR411**	**-**	**-**	
	[H:(2S)]			
	**ASP412**	**ASP412**	**-**	**-**

	[H:(1B); H-weak(1B) (9S); π:(2S)]	[H:(2B); H-weak: (2B) (4S); π:(2S)],		
	**-**	**MET415**	**MET415**	**MET415**
		[F:(6S)]	[H:(4B); π:(4S); H-weak:(4B) F:(5S); X:(1B)]	[H-weak:(6B); H:(1B)(S); π:(2S); F:(4S)]
	**-**	**THR416**	**-**	**-**
		[H:(1S)]		
	**VAL432**	**VAL432**	**-**	**-**
	[H-weak(3B); π:(2B) (1S)]	[H:(1B); H-weak:(2B)]		
	**-**	**-**	**THR433**	**-**
			Unclassified interaction	
	**-**	**-**	**GLN434**	**GLN434**
			[H:(3B)]	[H:(3B)]
	**LEU451**	**-**	**-**	**-**
	[H:(3B) one with 2bumps; H-weak(1B); π:(1S)]			
	**PRO452**	**-**	**-**	**-**
	[H:(1B); H-weak:(1B)]			
	**ASP454**	**-**	**-**	**-**
	[H:(2S); H-weak(3S); π:(7S)]			
	**SER457**	**-**	**-**	**-**
	[H:(1S); H-weak:(2B) (1S)]			
	**QLN460**	**-**	**-**	**GLN460**
	[H:(1S); H-weak:(2S)]			Unclassified interaction
	**-**	**ARG461**	**-**	**-**
		[H:(1S); F:(1S)]		
	**ARG464**	**-**	**-**	**ARG464**
	[H:(2S)]			[H:(1S)]
	**-**	**-**	**-**	**ARG467**
				Unclassified interaction
	**ARG481**	**-**	**-**	**-**
	[H:(7S)]			
	**-**	**PRO482**	**-**	**-**
		[H:(1B)]		
	**-**	**GLY484**	**-**	**-**
		[H:(4B)]		

	**-**	**MET485**	**-**	**-**
		[H:(2B); H-weak:(2B)		
	**ASP487**	**-**	**ASP487**	**ASP487**
	[H-Weak:(1S)]		[H:(1B)]	[H:(2B)]
	**-**	**-**	**-**	**GLU493**
				[H-weak:(1S); H:(1S)]
**SPARFLOXACIN**	**-**	**-**	**-**	**PRO230**
				[H:(1B); H-weak:(1B); F:(1S)]
	**-**	**THR269**	**-**	**-**
		[H:(1S)]		
	**-**	**GLY271**	**-**	**-**
		[H:(1B)]		
	**-**	**-**	**GLU291**	**GLU291**
			[H:(2S); H-weak:(1S)]	[π:(1S); H:(1S); F:(1S)]
	**HIS293**	**HIS293**	**-**	**HIS293**
	[H:(1B) with 2 bumps; H-weak:(2B)]	[π:(1S)]		[H:(1B); F:(1S)]
	**THR295**	**-**	**VAL295**	**GLN295**
	[H:(3B) one with bump (2S); H-weak(2S); π:(1B)]		[H:(2B) π:(6S), π-H:(2B); F:(7S); X:(1B)]	[H:(1B) (1S); H-weak:(1B) (2S); π:(2S)]
	**ASP296**	**-**	**ASP296**	**-**
	[H:(1S)]		[H:(1S); F:(2S); π:(1S); X:(1S)]	
	**-**	**SER297**	**-**	**-**
		[H:(2S)]		
	**-**	**THR298**	**-**	**-**
		[H:(3S); F:(1S); π:(1S)]		
	**ASP412**	**-**	**ASP412**	**ASP412**
	[H:(1B) (2S); H-weak:(3S); π:(2S)]		Bump	[H:(2S); H-weak:(1B) (1S)]
	**-**	**-**	**MET415**	**MET415**
			[H:(1B); H-weak:(4B); X:(1B)]	[π:(2S); F:(3S)]
	**-**	**THR416**	**-**	**-**
		Unclassified interaction		
	**VAL432**	**-**	**VAL432**	**-**
	[H:(1B); H-weak:(1B)]		[H:(1B); H-weak:(5B); X:(1B)]	

	**THR433**	**-**	**THR433**	**-**
	[H:(1S)]		[H:(1S)]	
	**GLN434**	**GLN434**	**GLN434**	**GLN434**
	Unclassified interaction	[H:(2B) (2S)]	[H:(1B); H-weak:(2B)]	[H:(5B); H-weak:(2B)]
	**LEU451**	**-**	**-**	**-**
	[H:(3B)]			
	**-**		**PRO452**	**-**
			Unclassified interaction	
	**-**	**-**	**ASP454**	**ASP454**
			[H:(1S); H-weak:(1S); X:(3S)]	[H:(3S)]
	**SER457**	**-**	**-**	**-**
	[H:(1B) (1S); H-weak:(1S); π:(1S)]			
	**GLN460**	**-**	**-**	**GLN460**
	[H:(2S); π:(1S)]			[H:(1B) (1S)]
	**ARG464**	**-**	**-**	**-**
	[H:(2S)]			
	**ARG467**	**-**	**-**	**ARG467**
	[H:(1S)]			[H:(2S)]
	**ARG481**	**ARG481**	**ARG481**	**ARG481**
	[H:(2S); π:(2S)]	[H:(3S); π:(1S)]	[H:(1S)]	[H:(2S)]
	**-**	**PRO482**	**-**	**-**
		[H:(1B)]		
	**GLY484**	**-**	**-**	**-**
	[H:(1B)]			
	**MET485**	**MET485**	**MET485**	**-**
	[H:(6B); H-weak(4B)]	[H:(1B)]	[H:(1B)]	
	**ASP487**	**-**	**ASP487**	**ASP487**
	[H:(1B)]		[H:(3B)]	[H:(3B); 2 bumps with side chain]
	**-**	**GLU493**	**-**	**GLU493**
		[H:(1B); H-weak:(4S)]		Unclassified interaction
	**-**	**TYR502**	**-**	**-**
		Unclassified interaction		
	**-**	**-**	**-**	**ASN556**
				[H:(1S)]
**PEFLOXACIN**	**-**	**-**	**ASN229**	**ASN229**
			[H:(2S)]	[H:(2S)]

	**-**	**-**	**-**	**SER231**
				1 bump
	**-**	**-**	**-**	**GLU291**
				[H-weak:(3S); H:(1S); F:(1S)]
	**HIS293**	**HIS293**	**HIS293**	**HIS293**
	[H:(1B)(1S); H-weak:(2S)]	[H:(1S); H-weak:(1B)(1S)]	[H:(2S); π-H:(1S); π:(2S); X:(1B)]	[H-weak:(1B)(3S); H:(1S); π:(3S); F:(2S)]
	**THR295**	**THR295**	**VAL295**	**GLN295**
	[H:(2B)(3S); H-weak:(2S)]	[H:(1B)(1S); H-weak:(5B)(1S)]	[H:(4B); π-H:(1B); π:(2S); F:(9S)]	[π:(S); F:(1B); H-weak:(1B)(3S); H:(3B)(S);]
	**ASP296**	**ASP296**	**-**	**-**
	Unclassified interaction	[H:(2S); π:(6S)]		
	**-**	**SER297**	**ALA297**	**-**
		[H:(1S); H-weak:(1S)]; one bump	[F:(1S)]	
	**-**	**-**	**-**	**THR298**
				Unclassified interaction
	**HIS369**	**-**	**-**	**-**
	[H:(1B); H-weak:(1B)]			
	**LYS371**	**-**	**-**	**-**
	[H:(1B); F:(1S); π:(1S)]			
	**THR411**	**-**	**-**	**-**
	[H:(2S); π:(1S)]			
	**-**	**ASP412**	**-**	**-**
		[H:(1S); H-weak:(2B); π:(2S)]		
	**-**	**-**	**MET415**	**MET415**
			[H:(2B); H-weak:(3B); F:(2S); π:(2S); X:(1B)]	[H-weak:(4B); H:(3B)(S); π:(4S); F:(6S)]
	**THR416**	**-**	**-**	**-**
	[H:(1S); H-weak:(1S)]			
	**VAL432**	**-**	**-**	**-**
	[H-weak:(4B); π:(2B)(1S); F:(1S)]			
	**-**	**GLN434**	**GLN434**	**GLN434**
		[H:(1B); H-weak:(1B)(3S)]	[H:(1B); π-H:(1B); X:(1S)]	[H:(2B)]
	**LEU451**	**-**	**-**	**-**

	[H:(2B); H-weak:(2B) one with bump; π:(1S)]			
	**ASP454**	**-**	**ASP454**	**ASP454**
	[H:(1S); H-weak:(1S); π:(6S)]		[H:(1S)]	[H-weak:(2S); H:(1S)]
	**SER457**	**-**	**-**	**-**
	[H:(3S); H-weak:(1B)(1S); π:(1S)]			
	**GLN460**	**GLN460**	**-**	**GLN460**
	Unclassified interaction	[H:(2S)]		[H:(2S)]
	**-**	**-**	**-**	**ARG464**
				[H:1S]
	**ARG481**	**-**	**ARG481**	**-**
	[H:(8S)]		[H:(2S)]	
	**-**	**GLY484**	**-**	**-**
		Unclassified interaction		
	**MET485**	**MET485**	**MET485**	**-**
	[H:(1B); H-weak:(1B)]	[H:(1B); H-weak:(2B)]	[H:(1B)]	
	**ASP487**	**-**	**ASP487**	**ASP487**
	[H:(1B); H-weak:(1S)]		[H:(2B)]	[H:(1B)]
	**-**	**SER489**	**-**	**-**
		Unclassified interaction		
	**-**	**GLU493**	**-**	**-**
		[H:(1B); H-weak:(2B)(1S)]		
	**-**	**TRP501**	**-**	**-**
		[H:(2S); H-weak:(1S); π:(2S)]		
	**-**	**ASP555**	**-**	**-**
		[H:(1B); H-weak:(1B)]		
